# School grades and educational attainments of adolescents and young adults born preterm

**DOI:** 10.1038/s41598-022-27295-4

**Published:** 2023-01-05

**Authors:** Suvi Alenius, Eero Kajantie, Reijo Sund, Markku Nurhonen, Peija Haaramo, Pieta Näsänen-Gilmore, Sakari Lemola, Katri Räikkönen, Daniel D. Schnitzlein, Dieter Wolke, Mika Gissler, Petteri Hovi

**Affiliations:** 1 Finnish Institute for Health and Welfare, Mannerheimintie 166, P.O. Box 30, 00271 Helsinki, Finland; 2 Children’s Hospital, University of Helsinki and Helsinki University Hospital, Helsinki, Finland; 3 Faculty of Medicine, PEDEGO Research Unit, MRC Oulu, Oulu University Hospital and University of Oulu, Oulu, Finland; 4 Department of Clinical and Molecular Medicine, Norwegian University of Science and Technology, Trondheim, Norway; 5 Faculty of Health Sciences, School of Medicine, Institute of Clinical Medicine, University of Eastern Finland, Kuopio, Finland; 6 Tampere Center for Child, Adolescent, and Maternal Health Research: Global Health Group, Faculty of Medicine, and Health Technology, Tampere University, Tampere, Finland; 7 Department of Psychology, Bielefeld University, Bielefeld, Germany; 8 Department of Psychology, University of Warwick, Coventry, UK; 9 Department of Psychology and Logopedics, Faculty of Medicine, University of Helsinki, Helsinki, Finland; 10 Institute of Labour Economics, Leibniz University, Hannover, Germany; 11 Institute of Labor Economics (IZA), Bonn, Germany; 12 Region Stockholm, Academic Primary Health Care Centre, Stockholm, Sweden & Karolinska Institute, Department of Molecular Medicine and Surgery, Stockholm, Sweden

**Keywords:** Epidemiology, Paediatric research

## Abstract

Attendance in special education (SE) is more common among individuals born preterm than among those born at term. Less is known about school grades of those born preterm in mainstream education (ME), and how these grades predict later educational attainment. This population-based register-linkage study assessed (1) attendance in SE, and then focused on those in ME by assessing (2) school grades at 16 year, (3) completed educational level at 25 year, and (4) school grades as predictors for completed education by gestational age (GA) with full-term birth (39–41 completed weeks) as reference. The sample comprised 223,744 individuals (10,521 preterm, 4.7%) born in Finland (1/1987–9/1990). Of the sample, 4.9% attended SE. Those born preterm had up to 5.5-fold rates for SE. In ME, those born extremely preterm (EPT) had marginally lower mathematics grades compared with full-term counterparts, whilst those born late preterm or early term had slightly higher grades. Those born EPT or very preterm had lower physical education grades in ME. However, the minor differences in school grades according to GA appear not to translate into educational differences in young adulthood. The associations between school grades at 16 year and completed education at 25 year did not vary by GA.

## Introduction

Annually 14.9 million live-born infants worldwide are born preterm (before 37 weeks’ gestation)^1^. Even more are born at 37–38 weeks (early term): they amount to 16–31% in high-income countries^2,3^. Both preterm and early term births impact disease risk and mortality^4–6^, and cause considerable population-level economic consequences^7,8^. For example, those born before 32 weeks of gestation, or at very (VLBW; < 1500 g) or extremely low-birthweight (< 1000 g) have on average lower educational attainments in childhood^9–14^, adolescence^9,12,13,15,16^, and young adulthood^9,11,17–20^ compared with those born at term. However, some contradicting results have been reported among those born very preterm^21^. When preterm birth is treated as a homogenous group, the study findings of three meta-analyses^22–24^ supplemented with two individual studies assessing those born at less than 37 weeks’ gestation^25,26^ are similar; individuals born preterm fare worse than their term born counterparts. Additionally, those born preterm^25,27–30^ or at low birth weight^15,16^ have more likely special educational needs, as do also moderately- or late preterm^31–33^, and even early term^34^ born individuals.

The association between the whole continuum of gestational age (GA) and educational attainments at adolescence^27,35–37^ and adulthood^38–40^ shows a trend for lower education with declining GA. However, we do not know whether this trend is explained by those who have special educational needs, or whether it is present also among those who attend mainstream education. Moreover, we know relatively little about whether school performance differ by school subject^41^, and little is known about how school grades predict educational attainment in adulthood. Hence, we had four main aims in assessing the later educational outcomes among those born preterm or early term. We (1) examined whether attending special education varied by GA. We then focused on those attending mainstream education and assessed (2) whether school grades at 16 years and (3) completed educational levels at 25 years varied according to GA. Finally, we examined (4) whether the associations between school grades at 16 years and completed education at 25 years varied by GA.

## Methods

### Data sources

The data sources include six nationwide administrative registers; (1) Finnish Medical Birth Register (MBR), (2) Central Population Register (CPR; updated through April 2012), (3) Register of Congenital Malformations (RCM, through January 2015), (4) The Finnish Care Register for Health Care (CRHC, through December 2015), (5) Statistics Finland’s registers (through December 2015) (including data based on the National Joint application Register maintained by the National Board of Education, from January 2003 through December 2007), and data on completed education (through December 2015), and (6) from the registers of the Social Insurance Institution of Finland (SII, through December 2015). These registers and their validity are described in Supplementary Methods and elsewhere^42,43^. Individual level register-linkages were done by encrypted personal identity codes (ePIC). The registered persons were not contacted. Based on Finnish and EU legislation, individual consents are not required in research based solely on analyzing pseudonymized register data if the registered persons are not contacted. The Coordinating Ethics Committee of the Hospital District of Helsinki and Uusimaa and applicable register authorities approved the study protocol which included the use of register data without need for individual consent, as allowed by Finnish and EU legislation. The study was conducted in accordance with the Declaration of Helsinki.

### Study population

**Figure 1:**
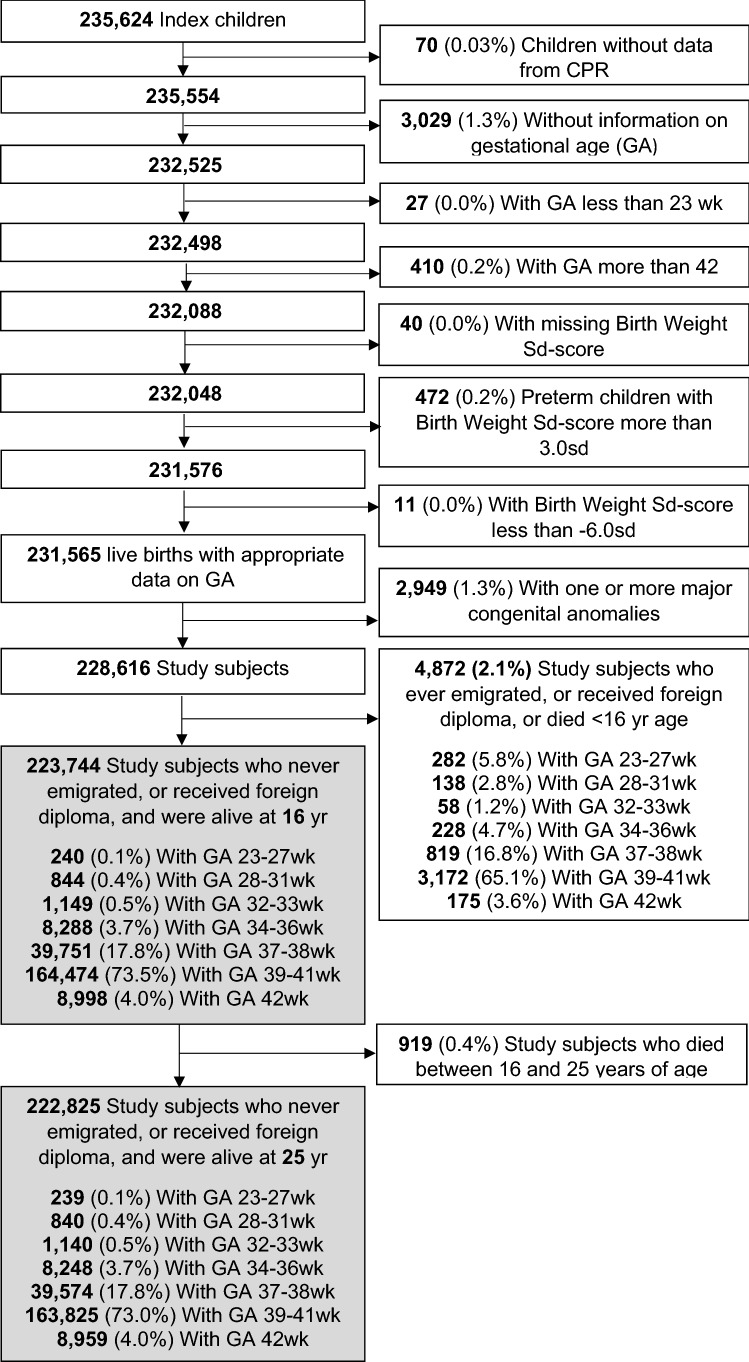
Study population. Note that information on the emigrations and deaths of the individuals were accessible only before 30th April 2012. Birth weight SD-score (BWSDS) was considered inaccurate if it was ≤ − 6.0, or, among preterm index children, > 3.0 according to national sex specific birth weight standards^44^ available for the gestational weeks of 23–43.

We identified from the MBR 235,624 index children with a valid PIC (99.8% of all live-born children) born in Finland between January 1, 1987, and September 30, 1990. After exclusions of a maximum of 26,909 (11.4%) individuals as illustrated in Fig. 1, we had 223,744 or 222,825 remaining, depending on the outcome, in our analyses. To take into account possible within-family influences and correlations we, in sensitivity analyses, included only each mother’s first child during the recruiting period (188,589, 84.3%).

### Exposure, covariates, and outcomes

#### Exposure

GA was assessed according to the best clinical estimate (based on ultrasound and/or maternal last menstrual period) and categorized as follows: extremely preterm; 23–27 full weeks, very preterm; 28–31 weeks, moderately preterm; 32–33 weeks, late preterm; 34–36 weeks, early term; 37–38 weeks, full-term; 39–41 weeks (reference), and post term; 42 weeks. In sensitivity analyses and in the analyses assessing the role of school grades on completed educational level the extremely, very, and moderately preterm groups were combined into ‘early preterm’ group.

#### Covariates

The covariates were added to the final model as four separate models built on each other. They included information on sex, parental ages, year of birth, parental highest attained educations, smoking in pregnancy, marital status at childbirth, birth order, the birth-weight-standard-deviation score (BWSDS), gestational disorder(s), and child severe medical condition (SMC).

The amount of missing data within the covariates included to the models was 0.0% for maternal age (n = 1), 0.0% for birth order (n = 13), 0.0% (n = 14) for maternal education, 0.4% (n = 997) for maternal marital status at childbirth, 1.1% (n = 2530) for paternal age, 1.1% for paternal education (n = 2530), and 1.9% (n = 4253) for maternal smoking in pregnancy. For categorical dummy-coded covariates missing data was coded to a separate category within each covariate, except missing maternal education (n = 14), missing maternal age (n = 1), and missing birth order (n = 13) which were coded to ‘basic or unknown education only’ category, the ‘20–34 year’ category, and to the ‘not first born’ category. Concerning the only continuous covariate, BWSDS, there were no missing values in the analyses.

The covariates were categorized or treated as continuous variable in the four models (1–4) as follows; (1) the sex (male vs. female), maternal age (< 20 year vs. 20–34 year vs. ≥ 35 year) and paternal age (< 20 year vs. 20–34 year vs. ≥ 35 year vs. unknown), the year of birth (1987 vs. 1988 vs. 1989 vs. 1990), (2) maternal highest attained education (basic or unknown education only *vs.* upper secondary less than tertiary *vs.* lower tertiary or more), paternal highest attained education (basic or unknown education only *vs.* upper secondary less than tertiary vs. lower tertiary or more vs. data on paternal educational level missing), (3) smoking in pregnancy (yes vs. no vs. unknown smoking status), marital status at childbirth (married *vs.* unmarried *vs*. unknown), the birth order (first born; yes vs. no), the BWSDS (continuous), gestational disorder(s) (gestational diabetes and/or gestational hypertension and/or intrahepatic cholestasis of pregnancy; yes vs. no), and (4) severe medical condition (yes vs. no).

The data on parental education, paternal age, and the birth dates on such siblings that shared the same mother with the individual included to the study originated from CPR, and were missing for such individuals who have no registered father in the CPR (n = 2530, 1.1%), or lacked a valid maternal personal identity code (n = 13, 0.0%) respectively. As birth order appear to have an association with one’s intelligence and educational attainments^45–47^, we computed a covariate (*first born* vs. *not first-born*) based on the birth dates of maternal previous live-born children (biological or adoptive). Data on disability allowances granted due to specific disability/disabilities or chronic illness originated from the register maintained by SII. Based on that data we formulated an aggregate variable reflecting the severe medical condition of the individual (SMC) before 16 years of age. This variable was computed following Moster et al.^39^ Major or other medical conditions were considered as present if the index child had received monetary benefit from the SII due to specific disability/disabilities or chronic illness presented in Supplementary Table S1.

Information on maternal age and marital status at the birth of the child, as well as data on maternal smoking in pregnancy came from the Medical Birth Register (MBR). The information on smoking during index pregnancy is recorded to MBR as it was self-reported at the first antenatal clinic appointment, which usually occurs at 9–10 completed weeks of gestation. The MBR served also as a source for the birth weight and dichotomous information on the sex of the individual. From the data on birth weight combined with the GA and the sex of the infant, the variables on the birth weight standard deviation score, BWSDS, (continuous) and on the smallness for GA status (*birth weight more than − 2 SDs below the mean/ birth weight more than − 2 SDs*) were derived by employing current national growth charts by Sankilampi et al.^44^ BWSDS as a continuous variable was included in the analysis as a proxy for fetal growth restriction, which appear to influence cognition and school performance^48^. Data on maternal pregnancy disorders were also drawn from the MBR and supplemented with data drawn from Finnish Care Register for Health Care (CRHC). A maternal pregnancy disorder was considered as present if any of the following three diagnoses appeared within the MBR or CRHC during the index pregnancy (from 20 weeks before expected date of delivery of the index child to 90 days after actual birth date): (1) gestational diabetes; *International Classification of Diseases, 8th Revision* (ICD-8) code 761.10, and *International Classification of Diseases, 9th Revision* (ICD-9) codes 6480A and 6488A, (2) maternal hypertensive disorder; *ICD-8* codes 637.01 and 637.03–637.99, or *ICD-9* codes 6420X-6429C, or (3) intrahepatic cholestasis of pregnancy; *ICD-8* codes 639.00–639.09, or *ICD-9* codes 6467A and 6467X^42^. We chose to include gestational diabetes, maternal hypertensive disorder, and intrahepatic cholestasis of pregnancy to this composite variable as these conditions are the most common such pregnancy related conditions that are treated and diagnosed at *in-hospital specialty care* and that are therefore derivable also from the CRCH allowing us to supplement the data originating from MBR.

Overall, the selection of covariates was mainly based on previous literature and data availability from the administrative registers, and partly on the univariate associations presented in the Supplementary Table S2. Such variables that were suggested as potential confounders in previous literature, but whose univariate association to the education at 25 years of age was not statistically significant were excluded from the models. The highest ever attained educational levels of both parents separately were selected as covariates to adjust for instead of choosing covariates that reflect educational levels at the birth of the index child or parental life course variations of education. This was done as we thought that the highest ever attained education of the parent(s) mirrors parental characteristics that are associated with the risk of preterm birth and the socioeconomic environment to which a person is exposed to during childhood and adolescence, and accordingly the parental role models, resources, and networks.

More detailed information on some of the covariates is available elsewhere^42,43^.

#### Outcomes

The Finnish education system (see Supplementary Methods) principally consists of a 9-year compulsory education between 7 and 16 years of age, after which a basic education diploma provides assessments on all school subjects (grading from 4 (fail) to 10 (excellent)). Completing compulsory education later than at 16 years may indicate later school start, longer preschool, repetition of school year, or voluntary additional basic education. The gradings are based on nationally defined criteria but not on standard tests and serve as selection criteria for further education. Of all gradings, the grade point average of all theoretical school subjects (native language, foreign languages, religion, history, mathematics, physics, chemistry, biology, and geography, but not arts, music, handicrafts, or physical education) is the selection criterion most often applied by the educational institutions.

Compulsory education is divided into mainstream education and special education (see Supplementary Methods). In special education individual assessment criteria are applied, hence individual grades are non-comparable. Moreover, attending special education may affect eligibility to certain post-compulsory education. In our work an individual was considered to have attended mainstream education if he/she had a diploma indicating no participation in special education in any of the school subjects.

The outcomes were: (1) the proportions of individuals in mainstream education, special education, and discontinued compulsory education (in subsequent analyses we included only students in mainstream education to ensure the comparability of the educational attainments); (2) school grades in mainstream education on mathematics, native language (Finnish, Swedish, or other), physical education, and the grade point average of all theoretical school subjects; (3) completed educational level at 25 years of age; and (4) the role of school grades on predicting completed educational level at 25 years.

### Statistical analyses

The only continuous variable in our work was birth weight standard deviation score (BWSDS), and its normality was checked by visual inspection i.e., plotting the data for detection of possible non-central or skewed distributions. No major departure from normality was observable. The proportions of individuals in mainstream education, special education and discontinued compulsory education were assessed by multinomial logistic regression, and school grades by ordinary linear regression models^49^. We trichotomized the highest completed educational level at 25 years according to ISCED (International Standard Classification of Education); (1) basic or unknown education only; ISCED level < 3, “low”, or (2) upper secondary, less than tertiary; ISCED 3–5, “intermediate” or (3) lower tertiary or more; ISCED 6–8), “high”, and assessed it by employing multinomial logistic regression models by having intermediate education as a reference.

Several separate regression models were employed when assessing the role of school grades on completed educational level at 25 year. Individuals included in the study were followed up from 16 years until the end of the year they reached the age of 25 years. School grades in three groups (4–6 low, 7–8 average, 9–10 high) served as potential moderators for the association between GA and education at 25 year (‘low’, or ‘high’ vs. ‘intermediate’), by having full-term category and grade category 7–8 as reference. Multinomial regression models provided Odds Ratios (OR) with 95% confidence intervals (CI). The ORs were considered to differ from 1.0 in a statistically significant manner if the 95% CI did not include 1.0. Interaction *P* values from comparisons of interaction and main-effects-only models were estimated.

In sensitivity analyses we included only the first child of each mother born during the recruiting period.

SPSS 27 was the statistical software.

## Results

Tables 1, 2 and 3 present the characteristics of 223,744 individuals (10,521, 4.7% preterm) and their parents. Supplementary Table S2 shows the association between the different covariates included in the models and education at 25 year within those who attended mainstream education and indicates that males are more likely to have basic education only at 25 year than females. However, as GA was associated with the attained education at 25 year similarly in both sexes, we report all results pooled.

**Table 1:** Characteristics of individuals included to the study by GA^a^ category.

	Extremely preterm	Very preterm	Moderately preterm	Late preterm	Early term	Full term	Post term	Total cohort
	23–27 weeks	28–31 weeks	32–33 weeks	34–36 weeks	37–38 weeks	39–41 weeks	42 weeks	23–42 weeks
**Index children**, n (%)	240 (0.1)	844 (0.3)	1149 (0.5)	8288 (3.7)	39,751 (17.8)	164,474 (73.5)	8998 (4.0)	223,744 (100.0)
Length of gestation; week, mean (SD)	26.4 (1.2)	30.4 (1.1)	33.1 (0.6)	35.9 (0.8)	38.2 (0.5)	40.3 (0.8)	42.2 (0.2)	39.8 (1.7)
Male, n (%)	130 (54.2)	486 (57.6)	630 (54.8)	4482 (54.1)	20,997 (52.8)	83,373 (50.7)	4653 (51.7)	114,751 (51.3)
Birth weight; g, mean (SD)	903 (179)	1454 (307)	1980 (390)	2701 (479)	3323 (479)	3682 (459)	3 861 (463)	3569 (550)
Birth weight SD score, mean (SD)	0.26 (1.29)	− 0.16 (1.49)	− 0.30 (1.48)	− 0.17 (1.29)	− 0.00 (1.15)	0.03 (1.02)	− 0.03 (1.01)	0.01 (1.06)
Small for gestational age; SGA, n (%)	11 (4.6)	110 (13.0)	158 (13.8)	675 (8.1)	1442 (3.6)	3188 (1.9)	201 (2.2)	5785 (2.6)
Twins, triplets, or quadruplets, n (%)^b^	47 (19.6)	197 (23.3)	252 (21.9)	1421 (17.1)	2255 (5.7)	641 (0.4)	0 (0.0)	4813 (2.2)
Medical disability, n (%)^c^	97 (40.4)	194 (23.0)	133 (11.6)	530 (6.4)	1941 (4.9)	6945 (4.2)	445 (4.9)	10,285 (4.6)
Other major disabilities, n (%)^c^	23 (9.6)	25 (3.0)	24 (2.1)	85 (1.0)	238 (0.6)	897 (0.5)	54 (0.6)	1346 (0.6)
First born^d^	100 (41.7)	433 (51.3)	586 (51.4)	3802 (45.9)	15,173 (38.2)	64,477 (39.2)	4784 (53.2)	89,355 (39.9)
**Birth year, n (%)**								
1987	62 (25.8)	223 (26.4)	278 (24.2)	2092 (25.2)	9803 (24.7)	41,803 (25.4)	2156 (24.0)	56,417 (25.2)
1988	75 (31.3)	194 (23.0)	307 (26.7)	2192 (26.4)	11,123 (28.0)	43,907 (26.7)	2155 (23.9)	59,953 (26.8)
1989	57 (23.8)	247 (29.3)	304 (26.5)	2257 (27.2)	10,584 (26.6)	43,926 (26.7)	2575 (28.6)	59,950 (26.8)
1 January–30 September 1990	46 (19.2)	180 (21.3)	260 (22.6)	1747 (21.1)	8241 (20.7)	34,838 (21.2)	2112 (23.5)	47,424 (21.2)

^a^GA—gestational age. Completed weeks of gestation.

^b^A total of 163 children were triplets etc.

^c^Following Moster et al.^39^ In the models Medical disability and Other major disabilities are combined to a one variable; Severe medical condition. The variable includes the following diagnosis groups or separate diagnoses: cerebral palsy, mental retardation, schizophrenia, disorders of psychological development behavior and emotion, epilepsy, blindness or low vision, and hearing loss. A complete list of diagnosis codes included in this variable is available from Supplementary table S1. Note that one individual may have more than one diagnosis.

^d^A total of 13 children missed data on birth order.

**Table 2:** Characteristics of the biological mothers of the individuals included to the study by GA^a^ category.

	Extremely preterm	Very preterm	Moderately preterm	Late preterm	Early term	Full term	Post Term	Total cohort
	23–27 weeks	28–31 weeks	32–33 weeks	34–36 weeks	37–38 weeks	39–41 weeks	42 weeks	23–42 weeks
Mother married at the birth of the index child, n (%)	166 (69.2)	588 (69.7)	830 (72.2)	6244 (75.3)	31,411 (79.0)	129,839 (78.9)	6672 (74.1)	175,750 (78.5)
Mother smoked during pregnancy, n (%)	52 (21.7)	148 (17.5)	237 (20.6)	1515 (18.3)	6239 (15.7)	23,803 (14.5)	1466 (16.3)	33,460 (15.0)
Maternal pregnancy disorder during index pregnancy, n (%)^b^	28 (11.7)	204 (24.2)	310 (27.0)	1715 (20.7)	6560 (16.5)	12,964 (7.9)	418 (4.6)	22,199 (9.9)
Maternal data available from the CPR, n	239	844	1149	8287	39,750	164,466	8996	223,731
Maternal age; years, mean (SD)	30.0 (5.5)	29.0 (5.7)	29.1 (5.9)	28.8 (5.6)	28.9 (5.4)	28.4 (5.1)	27.8 (4.9)	28.5 (5.2)
**Age**								
Less than 20 years, n (%)	4 (1.7)	37 (4.4)	49 (4.3)	319 (3.8)	1186 (3.0)	4569 (2.8)	322 (3.6)	6486 (2.9)
35 years or more, n (%)	51 (21.3)	160 (19.0)	221 (19.2)	1414 (17.1)	6374 (16.0)	20,640 (12.5)	876 (9.7)	29,736 (13.3)
**Maternal educational level, highest ever attained, n (%)**								
Basic only or unknown	47 (19.6)	130 (15.4)	231 (20.1)	1380 (16.7)	5966 (15.0)	21,172 (12.9)	1202 (13.4)	30,128 (13.5)
Upper-secondary, less than tertiary	152 (63.3)	569 (67.4)	723 (62.9)	5464 (65.9)	26,175 (65.8)	111,122 (67.6)	6069 (67.4)	150,274 (67.2)
Lower tertiary or more	41 (17.1)	145 (17.2)	195 (17.0)	1444 (17.4)	7610 (19.1)	32,180 (19.6)	1727 (19.2)	43,342 (19.4)

The amount of missing data was 0.0% (n = 14) for maternal education, 0.4% (n = 997) for maternal marital status at childbirth, and 1.9% (n = 4253) for maternal smoking in pregnancy.

^a^GA—gestational age. Completed weeks of gestation.

^b^Pregnancy disorder includes gestational diabetes, gestational hypertensive disorder, and intrahepatic cholestasis of pregnancy. A complete list of diagnose codes included in this variable is available from Supplementary Material and elsewhere^42^.

**Table 3:** Characteristics of the registered fathers of individuals included to the study by GA^a^ category.

	Extremely preterm	Very preterm	Moderately preterm	Late preterm	Early term	Full term	Post term	Total cohort
	23–27 weeks	28–31 weeks	32–33 weeks	34–36 weeks	37–38 weeks	39–41 weeks	42 weeks	23–42 weeks
Paternal data available from the CPR, n	236	822	1120	8124	39,233	162,804	8875	221,214
Paternal age; years, mean (SD)	32.4 (6.7)	31.2 (6.4)	31.3 (6.4)	31.1 (6.1)	31.2 (6.0)	30.8 (5.7)	30.4 (5.6)	30.9 (5.7)
**Age**								
Less than 20 years, n (%)	16 (1.5)^b^		17 (1.5)	76 (0.9)	272 (0.7)	1061 (0.6)	68 (0.8)	1510 (0.7)
35 years or more, n (%)	91 (37.9)	232 (27.5)	304 (26.5)	2191 (26.4)	10,398 (26.2)	38,009 (23.1)	1900 (21.1)	53,125 (23.7)
**Paternal educational level, highest ever attained, n (%)**								
Basic only or unknown	58 (24.2)	204 (24.2)	292 (25.4)	2009 (24.2)	9050 (22.8)	34,825 (21.2)	1998 (22.2)	48,436 (21.6)
Upper-secondary, less than tertiary	136 (56.7)	495 (58.6)	636 (55.4)	4783 (57.7)	22,972 (57.8)	97,626 (59.4)	5268 (58.5)	131,916 (59.0)
Lower tertiary or more	42 (17.5)	123 (14.6)	192 (16.7)	1332 (16.1)	7211 (18.1)	30,353 (18.5)	1609 (17.9)	40,862 (18.3)

The amount of missing data was 1.1% (n = 2,530) for paternal age and paternal education.

^a^GA—gestational age. Completed weeks of gestation.

^b^Gestational age categories of 23–31 weeks are combined because privacy regulations prevent us to display cell counts of three or less.

In mainstream education 94.9% of the students completed compulsory education within the same year they turned 16 year. Those born preterm completed compulsory education more often at 17 years of age; 7.0% vs. 3.9% in total cohort.

### Type of education in compulsory school

**Figure 2:**
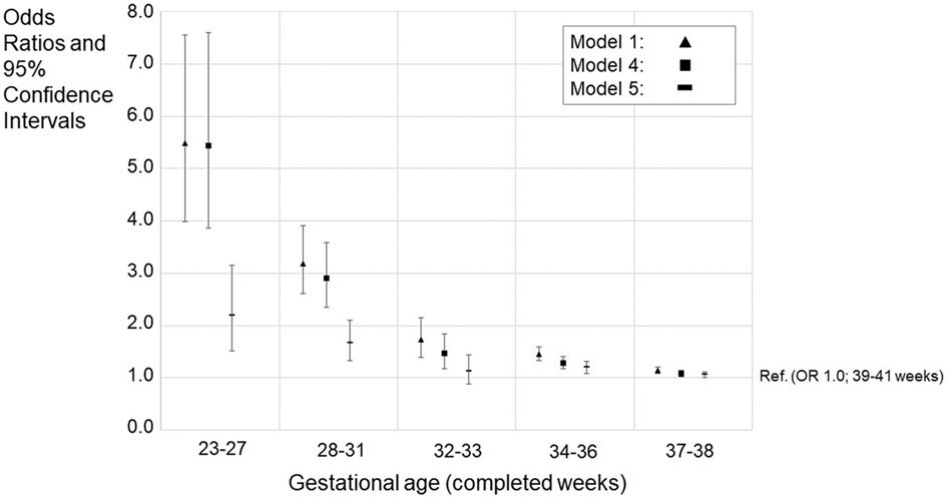
Odds ratios (ORs) and 95% confidence intervals (CIs) for special education in compulsory education according to gestational age category. The figure shows models 1, 4, and 5. Models 2 and 3 are available in the Supplementary Table 3. GA category 39–41 weeks is the reference group (OR = 1.0). Model 1; Unadjusted model, Model 4; Adjusted for the sex, birth year, maternal and paternal ages, maternal and paternal highest attained education, BWSDS, gestational disorder(s), maternal smoking at pregnancy, maternal marital status at the childbirth, and birth order, Model 5; Adjusted as Model 4, and for severe medical condition.

A total of 4.6% of those born full-term had attended special education compared to 20.0% of those born extremely preterm. The corresponding unadjusted percentages for other GA categories before full-term birth were as follows: very preterm 13.0%, moderately preterm 7.6%, late preterm 6.5% and early term 5.3%. Unadjusted OR for special education varied from 5.48 (95% CI 3.98–7.55) among extremely preterm individuals to 1.45 (95% CI 1.33–1.59) for those born late preterm and 1.15 (95% CI 1.09–1.21) for those born early term. The ORs for special education in all GA categories attenuated only slightly after adjustment for other covariates than severe medical condition (Model 4, Supplementary Table S3, Fig. 2). However, in the fully adjusted model, which included also severe medical condition, the OR varied from 2.19 (95% CI 1.52–3.16) among extremely preterm born adolescents to 1.20 (95% CI 1.09–1.32) for those born late preterm and 1.07 (95% CI 1.01–1.12) for those born early term, while the association between moderately preterm birth and special education attenuated being no longer statistically significant (*P* = 0.314) (Model 5, Supplementary Table S3, Fig. 2).

Severe medical condition of the individual, maternal smoking, and low parental ages and education were risk factors for discontinued compulsory education (see Supplementary analyses), but GA was not. In models adjusted for all the covariates those born late preterm had slightly lower risk for discontinued compulsory education (Supplementary Table S4, Supplementary Figure S1). A total of 3.5% of those born at 23–33 weeks’ gestation had unknown type of education (i.e., other than special or mainstream education or discontinuing school attendance) as compared to 1.5% of their full-term born peers. The corresponding percentages for those born late preterm or early term were 2.2% and 1.6% respectively.

### Grades at the end of compulsory school

**Figure 3:**
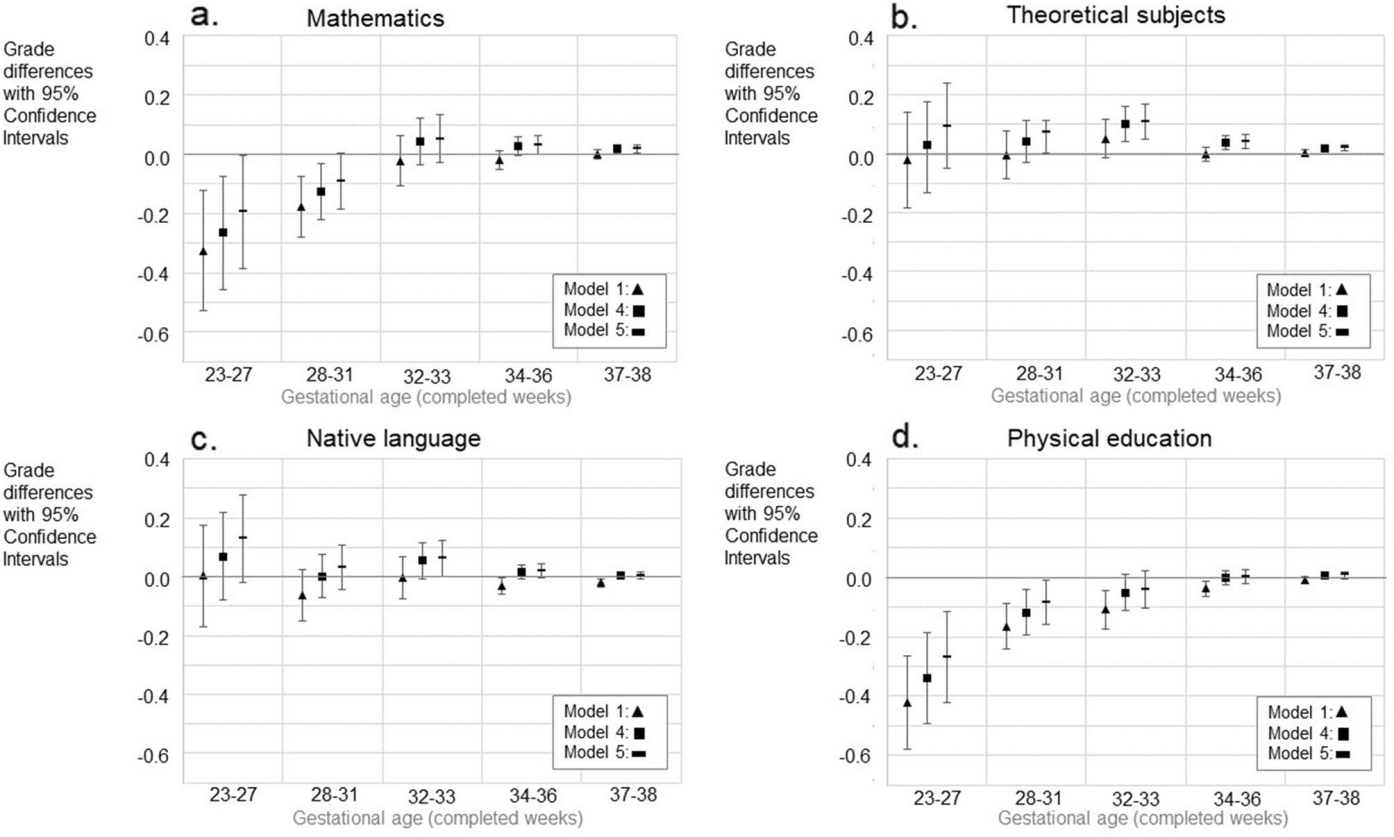
Differences in school grades (**a–d**) in mainstream education with 95% confidence intervals (CIs). The figures show models 1, 4, and 5. Models 2 and 3 are available in the Supplementary Tables 5–8. GA category 39–41 weeks is the reference group (with grade difference 0.0). Only such individuals who attended mainstream education in compulsory education are included. Model 1; Unadjusted model, Model 4; Adjusted for the sex, birth year, maternal and paternal ages, maternal and paternal highest attained education, BWSDS, gestational disorder(s), maternal smoking at pregnancy, maternal marital status at the childbirth, and birth order, Model 5; Adjusted as Model 4, and for severe medical condition.

Figure 3 and Supplementary Tables S5–S8 illustrate the grades (as grade differences in points and as z-scores) on mathematics, theoretical subjects, native language, and physical education in mainstream education by GA. In fully adjusted model those born extremely preterm had 0.2 (SD − 0.4 to 0.0) points lower mathematics grades than those born full-term, whilst those born late preterm or early term had in fully adjusted model slightly higher grades; 0.03 (SD 0.00–0.06) points and 0.02 (SD 0.00–0.03) points respectively. Very-, moderately- and early preterm births, as well as early term births were associated with marginally higher grades in theoretical subjects in fully adjusted models: (0.07 (SD 0.00–0.11) points among those born very preterm; 0.11 (SD 0.05–0.17) points among those born moderately preterm; 0.04 (SD 0.02–0.06) points; and 0.02 (SD 0.01–0.03) points among those born early term)). Moderately preterm birth was associated to 0.06 (SD 0.00–0.12) higher grade in native language, while those born extremely or very preterm had lower PE grades than those born full-term; − 0.26 (SD − 0.41 to − 0.11) and − 0.08 (SD − 0.15 to − 0.01) points respectively in fully adjusted models.

### Educational attainment at 25 years of age

**Figure 4:**
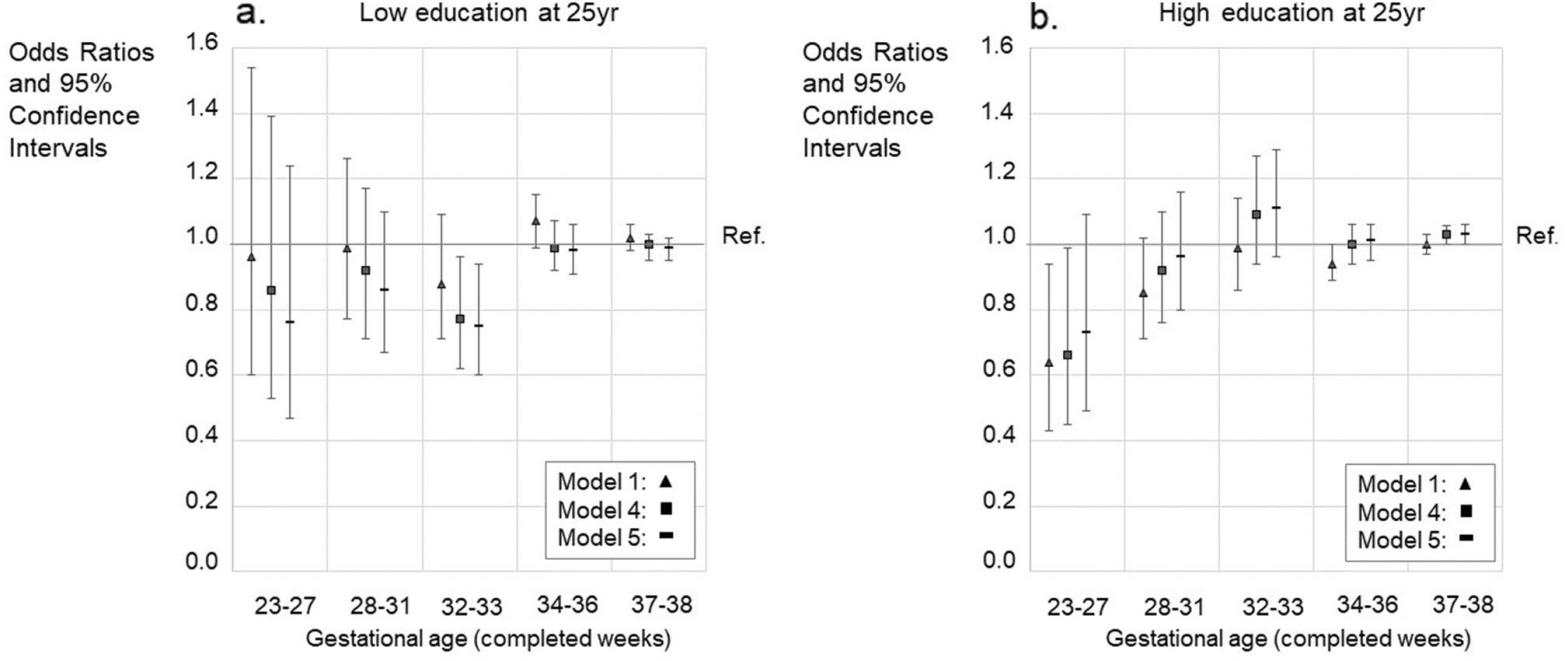
Education at 25 years of age. Intermediate education (upper secondary, less than tertiary) as a reference. **A** - Odds ratios (OR) with 95% confidence intervals for low education (basic only or unknown). **B** - Odds ratios (OR) with 95% confidence intervals for high (lower tertiary or more) education. The figure shows models 1, 4, and 5. Models 2 and 3 are available in the Supplementary Table 9ab. GA category 39–41 weeks is the reference group (with OR = 1.0). Only such individuals who attended mainstream education in compulsory education are included. Model 1; Unadjusted model, Model 4; Adjusted for the sex, birth year, maternal and paternal ages, maternal and paternal highest attained education, BWSDS, gestational disorder(s), maternal smoking at pregnancy, maternal marital status at the childbirth, and birth order, Model 5; Adjusted as Model 4, and for severe medical condition.

Multinomial logistic regression analyses estimated ORs for ‘low’ or ‘high’ education, ‘intermediate’ education being the reference. In unadjusted models those born extremely preterm were 0.6-fold (OR 0.64 (95% CI 0.43–0.94)) less likely to attain high education at 25 year than those born full-term. In such an unadjusted model the odds were also lower for those born late preterm (OR 0.94 (95% CI 0.89–1.00)). For other preterm GA categories, there were no differences in attaining high education according to the unadjusted model. In the fully adjusted model there were no differences between any of the preterm GA categories and attainment of high education. Those born moderately preterm had, however, an OR of 0.75 (95% CI 0.60–0.94) for low education at 25 year in a fully adjusted model. For other preterm GA categories, there were no differences in attaining low education as compared to those born full-term according to such a model (Fig. 4, Supplementary Table S9ab).

### The effect of school grades on completed education at 25 years of age

**Figure 5:**
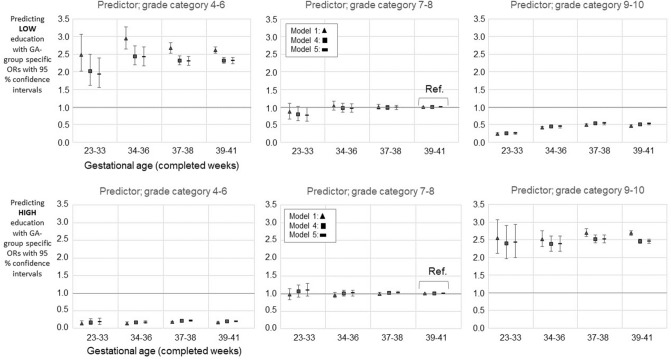
Mathematics grade and gestational age category together predicting [LOW] ‘basic only or unknown’ (upper panel) and [HIGH] ‘lower tertiary or more’ (lower panel) education. Comparisons to intermediate education i.e., ‘upper secondary, less than tertiary’. GA 39–41 and grade category 7–8 serves as a reference group. The figures show models 1, 4, and 5. Models 2 and 3 are available in the Supplementary Table 10ab. The *p* values (for Model 5) from the comparisons of interaction- and main-effects-only models were 0.721 for grade 4–6 group; 0.638 for grade 7–8 group; and 0.718 for grade 9–10 group. For unadjusted model (Model 1) the *p* values from the comparisons were as follows: 0.599 for grade 4–6 group; 0.785 for grade 7–8 group; and 0.704 for grade 9–10 group. Only such individuals who attended mainstream education in compulsory education are included. Model 1; Unadjusted model, Model 4; Adjusted for the sex, birth year, maternal and paternal ages, maternal and paternal highest attained education, BWSDS, gestational disorder(s), maternal smoking at pregnancy, maternal marital status at the childbirth, and birth order, Model 5; Adjusted as Model 4, and for severe medical condition.

Grades in mathematics, theoretical subjects, native languages, and physical education predicted completed educational level at 25 years independent of GA; School grade*GA specific interaction *P* values were non-significant in unadjusted models, and in models including all the covariates, i.e. fully adjusted models (Fig. 5, Supplementary Figures S2–S4, Supplementary Tables S10ab–S13ab).

### Sensitivity analyses

Including only each mother’s first-born child within the cohort years did not affect the interpretations of the results (data not shown).

## Discussion

We studied over 220,000 individuals and found that those born before 39 completed weeks’ gestation more likely attend special education during compulsory education. Among those in mainstream education, preterm birth appeared to be associated with marginally decreased grades in mathematics, and in physical education, but not in native languages or in theoretical subjects at 16 years of age. Among those who attended mainstream education, gestational age (GA) was only marginally associated with completed educational level at 25 years. Further, the grades at the end of compulsory education predicted the educational level similarly regardless of the GA. Our results indicate that the minor educational differences in mathematics and physical education grades according to GA in compulsory education did not lead to a greater gap in educational attainments later in young adulthood. While parental education was strongly associated with offspring education, these associations between GA and the outcomes were largely similar regardless of parental education.

Our study employs a whole population cohort with minimal loss to follow-up. It quantifies educational outcomes at two different time points and assesses the educational trajectories between these two. Register-linkages enabled by PICs provide reliable measures of academic performance at adolescence and young adulthood. Study results focusing on those who attended mainstream education are applicable to most born at suboptimal gestational age; even of those born extremely preterm, more than 70% attended mainstream education.

As to limitations we lacked data on whether the GA estimation was based on fetal ultrasound or on the last maternal menstrual period (LMP). LMP method may overestimate the GA. As fetal ultrasound was only being introduced in clinical practice in Finland in 1987–1990, the GA estimates may have moved towards a minor underestimation of preterm birth^50^, which would only have a trivial effect on our estimation. Further, information on emigrations and deaths after April 2012 were inaccessible. The magnitude of the bias caused by this can be considered as minor and lead to slight overestimation of the rates of low education and to minor imprecision in defining the study cohort; Statistics Finland’s aggregate data indicate mortality rate of only 65/100,000 and emigration rate of 808/100,000 among Finns aged 25–34 in 2019^51–53^. We also lacked data on such pre- and postnatal treatments that were rare during cohort birth years but are common in clinical practice nowadays. Improvements in the care of newborn infants during the recent decades may impact the generalizability of our results to those born at later years. Further, we lacked data on educational attainments at the end of compulsory education for those who did not apply for further education during 2003–2007. However, during the years 2004–2006 only 2.0% of students did not directly apply further education after completing compulsory education^54^. In addition, the cohort was only followed-up to 25 years age when some young adults may have their higher education still ongoing, whereas most of them would have completed their upper secondary education. Therefore, the results may partially reflect slower tempo in completing higher education. Lastly, the generalizability of the study findings to other national contexts may be affected by the differences between the school systems, especially outside the Nordic countries which share several but not all common traits in their educational systems^55^. As the gradings at the end of compulsory education in Finland are given by teacher and based on nationally defined criteria but not on standard test, they may be affected by teacher’s personal perceptions and preferences^56^.

Our results align with previous findings on more frequent special educational needs not only among those born most preterm, e.g., at extremely low birth weight^15,16^ but also among all children born before 39 completed weeks of gestation^25,27–33^. In our study, the robustness of the estimates combined with dose–response relationship between the declining GA and increasing rate of special education may indicate a developmental interference component associated to this tendency.

Previous meta-analyses on math and reading skills at any age of the individual show that those born preterm fare worse than those born at term^9,13,22–24^ in particular in mathematics^24^. These results do not align those of ours, showing only minor differences in mathematics as found in other studies among preterm born population with stringent control of confounders^57^, and no differences in native language grades by GA. However, as the studies included to these meta-analyses present assessments within a wide age range, have varying definitions for term birth, and especially as the majority of them appear to include also those who attended special education, our study may present more optimistic estimates as compared to theirs. A smaller Finnish study reported similar results to ours: very preterm born individuals without major disabilities performed similarly or better at 16 years as compared to those born at term^21^. A recent study of over 70,000 adolescents of 16–17 years found no differences in math or English language grades in California among those born moderately to late preterm as compared to those born at term^58^. In our study the marginal differences in estimates between native language and mathematics may be explained by the fact that some catch up with age in reading skills exists especially among those born at extremely or very low birth weight^15,59^, but not to the same extent in mathematics^24^.

As regards to the grades on theoretical subjects combined, we are unaware of previous studies. However, a Swedish register study^35^, with definition of 40–41 weeks for full-term birth, imply that grade averages of preterm children at 16 years of age were below those of full-term counterparts in mainstream education. We found essentially no differences in mean grades of theoretical subjects according to the GA. In some GA groups in some of the adjusted models, mean grades were even marginally higher compared with those born at term. Previous work also indicate that the association between declining GA and poorer school performance and lower IQ at adolescence are attributable to factors other than prematurity^36^, such as parental socioeconomic position^16,37^. The results of ours indicating lower grades on physical education among those born preterm may mirror the findings on motor impairments^60^, lower physical fitness^61^, and less leisure time physical activity^62^ among preterm born individuals.

Previous literature on educational attainments in adulthood, mainly also including those who attended special education, illustrates a trend for poorer academic performance with declining GA^38–40,63^. Our study shows that in mainstream education, preterm birth is not associated with higher risk of low education at 25 years, neither to a noticeably extent to lower likelihood for high educational level as compared to intermediate education.

Previous studies indicate that some positive impact of physical education on later academic achievements may exist^64,65^. Otherwise, we are not aware of previous studies assessing the trajectories of academic attainments from adolescence to young adulthood, nor across the whole range of GA. We found no differences in the impacts of different school grades on completed educational attainments at 25 years of age according to GA. This indicates that the minor education gap among those born most preterm as compared to those born full-term in mainstream education in compulsory education appear not to amplify in higher education and can be interpreted as supportive information to most families with preterm born children. However, even in mainstream education those born extremely preterm or very preterm still have lower grades in mathematics and physical education, and needing special education is inversely related to decreasing GA across the whole range of gestation from 38 to 23 completed weeks and affects eligibility to post-compulsory education.

In Finland developmental follow-up of all children is provided up to the pre-school age, and speech and occupational therapy are accessible during the kindergarten and pre-school period when needed. Further, in compulsory education support in form of school health care (including free access to school physicians, nurses, and psychologists), remedial assistance, student counseling, and visits to school social worker are available, and may have impact on the beneficial educational outcomes those born at suboptimal GA, even in special education.

## Conclusions

Children born preterm are more likely to attend special education with the highest rates seen at the lowest gestational ages. In mainstream education preterm born individuals have somewhat lower grades in mathematics and physical education, whilst achievement is similar to those born full-term in other school grades. Gestational age is not appreciably associated with educational level at 25 years of age. The school grades in mathematics, native language, physical education, and theoretical subjects, seem to predict completed educational level at 25 years regardless of the gestational age of the individual indicating that educational gap in adolescence appear not to widen in young adulthood.

## Supplementary Information

Supplementary Information.

## Data Availability

The datasets will not be made publicly available, even though the data are anonymized. Only members of the current study groups were granted access to the sensitive individual level data from the relevant registers. Further access rights are subject to permission from the registers: interested researchers may apply for data access rights from the Social and Health Data Permit Authority, Findata (https://findata.fi/en/). All relevant analysis results are shared and published in this article.

## Abbreviations

CI: Confidence interval
CPR: Central Population Register
CRHC: Finnish Care Register for Health Care
ELBW: Extremely low birthweight
GA: Gestational age
MBR: Finnish Medical Birth Register
OR: Odds ratio
RCM: Register of Congenital Malformations
SD: Standard deviation
SDS: Standard deviation score
SMC: Severe medical condition
SII: Social Insurance Institution
THL: Finnish Institute for Health and Welfare
VLBW: Very low birthweight
