# Author Correction: School grades and educational attainments of adolescents and young adults born preterm

**DOI:** 10.1038/s41598-023-30739-0

**Published:** 2023-03-06

**Authors:** Suvi Alenius, Eero Kajantie, Reijo Sund, Markku Nurhonen, Peija Haaramo, Pieta Näsänen-Gilmore, Sakari Lemola, Katri Räikkönen, Daniel D. Schnitzlein, Dieter Wolke, Mika Gissler, Petteri Hovi

**Affiliations:** 1 Finnish Institute for Health and Welfare, Mannerheimintie 166, P.O. Box 30, 00271 Helsinki, Finland; 2 Children’s Hospital, University of Helsinki and Helsinki University Hospital, Helsinki, Finland; 3 Faculty of Medicine, PEDEGO Research Unit, MRC Oulu, Oulu University Hospital and University of Oulu, Oulu, Finland; 4 Department of Clinical and Molecular Medicine, Norwegian University of Science and Technology, Trondheim, Norway; 5 Faculty of Health Sciences, School of Medicine, Institute of Clinical Medicine, University of Eastern Finland, Kuopio, Finland; 6 Tampere Center for Child, Adolescent, and Maternal Health Research: Global Health Group, Faculty of Medicine, and Health Technology, Tampere University, Tampere, Finland; 7 Department of Psychology, Bielefeld University, Bielefeld, Germany; 8 Department of Psychology, University of Warwick, Coventry, UK; 9 Department of Psychology and Logopedics, Faculty of Medicine, University of Helsinki, Helsinki, Finland; 10 Institute of Labour Economics, Leibniz University, Hannover, Germany; 11 Institute of Labor Economics (IZA), Bonn, Germany; 12 Region Stockholm, Academic Primary Health Care Centre, Stockholm, Sweden & Karolinska Institute, Department of Molecular Medicine and Surgery, Stockholm, Sweden

Correction to: *Scientific Reports* https://doi.org/10.1038/s41598-022-27295-4, published online 05 January 2023

The original version of this Article contained an error in Figures 2, 3, 4 and 5, where the legend for the visual cues incorrectly states ‘Model 2’ for the black square.

The correct legends now read:


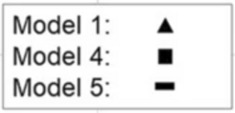


The original Figures 2, 3, 4 and 5 and their respective accompanying legend appear below.

The original Article has been corrected.

**Figure 2:**
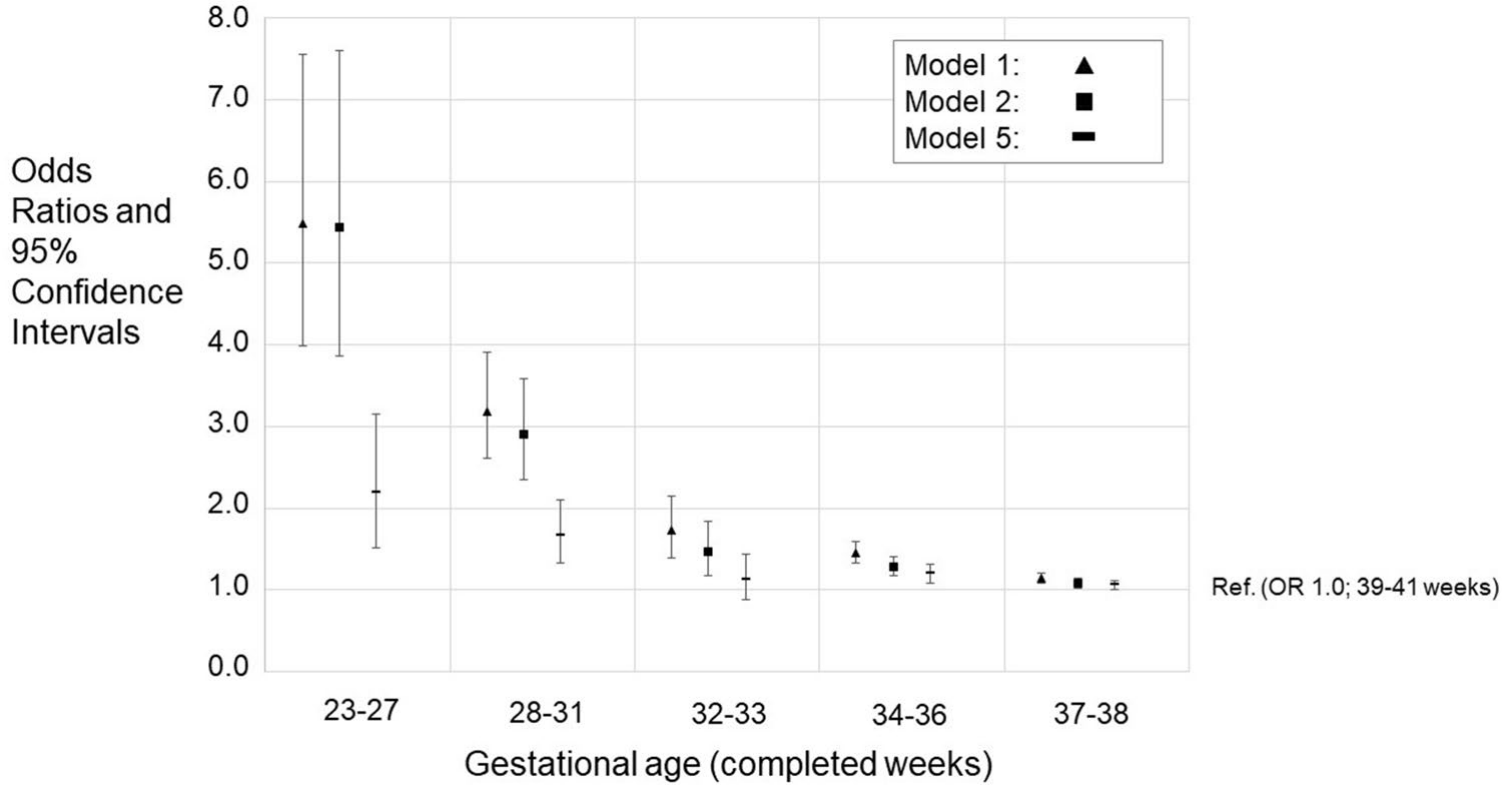
Odds ratios (ORs) and 95% confidence intervals (CIs) for special education in compulsory education according to gestational age category. The figure shows models 1, 4, and 5. Models 2 and 3 are available in the Supplementary Table 3. GA category 39–41 weeks is the reference group (OR = 1.0). Model 1; Unadjusted model, Model 4; Adjusted for the sex, birth year, maternal and paternal ages, maternal and paternal highest attained education, BWSDS, gestational disorder(s), maternal smoking at pregnancy, maternal marital status at the childbirth, and birth order, Model 5; Adjusted as Model 4, and for severe medical condition.

**Figure 3:**
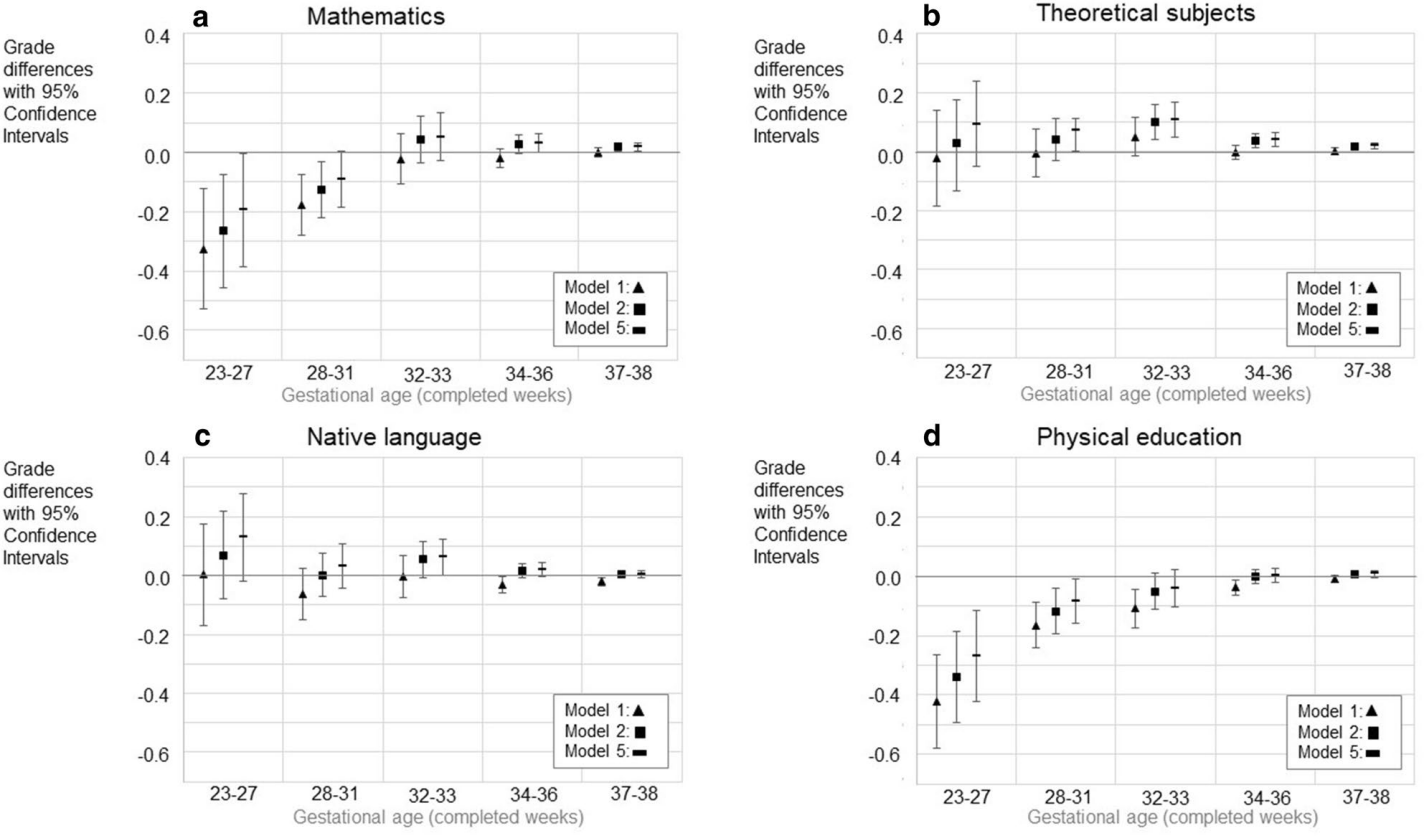
Differences in school grades (**a**–**d**) in mainstream education with 95% confidence intervals (CIs). The figures show models 1, 4, and 5. Models 2 and 3 are available in the Supplementary Tables 5–8. GA category 39–41 weeks is the reference group (with grade difference 0.0). Only such individuals who attended mainstream education in compulsory education are included. Model 1; Unadjusted model, Model 4; Adjusted for the sex, birth year, maternal and paternal ages, maternal and paternal highest attained education, BWSDS, gestational disorder(s), maternal smoking at pregnancy, maternal marital status at the childbirth, and birth order, Model 5; Adjusted as Model 4, and for severe medical condition.

**Figure 4:**
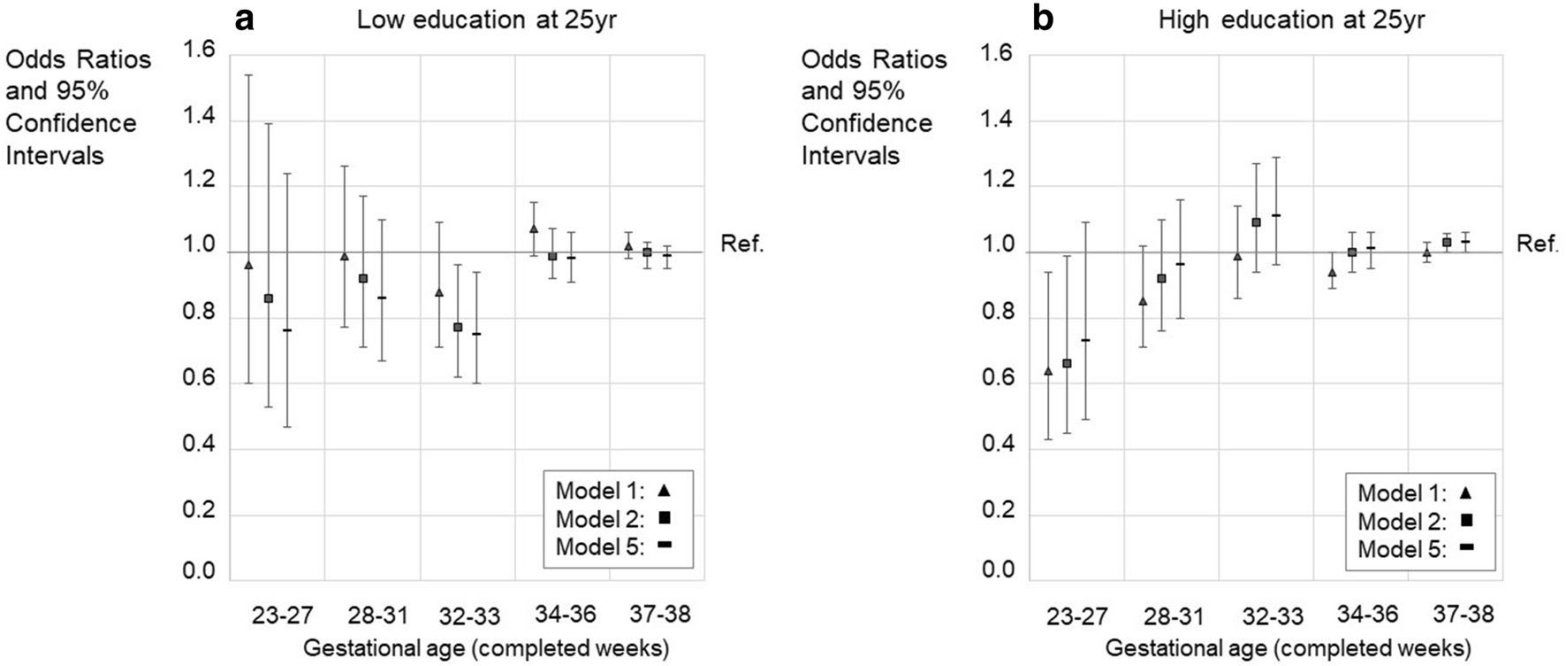
Education at 25 years of age. Intermediate education (upper secondary, less than tertiary) as a reference. **A** - Odds ratios (OR) with 95% confidence intervals for low education (basic only or unknown). **B** - Odds ratios (OR) with 95% confidence intervals for high (lower tertiary or more) education. The figure shows models 1, 4, and 5. Models 2 and 3 are available in the Supplementary Table 9ab. GA category 39–41 weeks is the reference group (with OR = 1.0). Only such individuals who attended mainstream education in compulsory education are included. Model 1; Unadjusted model, Model 4; Adjusted for the sex, birth year, maternal and paternal ages, maternal and paternal highest attained education, BWSDS, gestational disorder(s), maternal smoking at pregnancy, maternal marital status at the childbirth, and birth order, Model 5; Adjusted as Model 4, and for severe medical condition.

**Figure 5:**
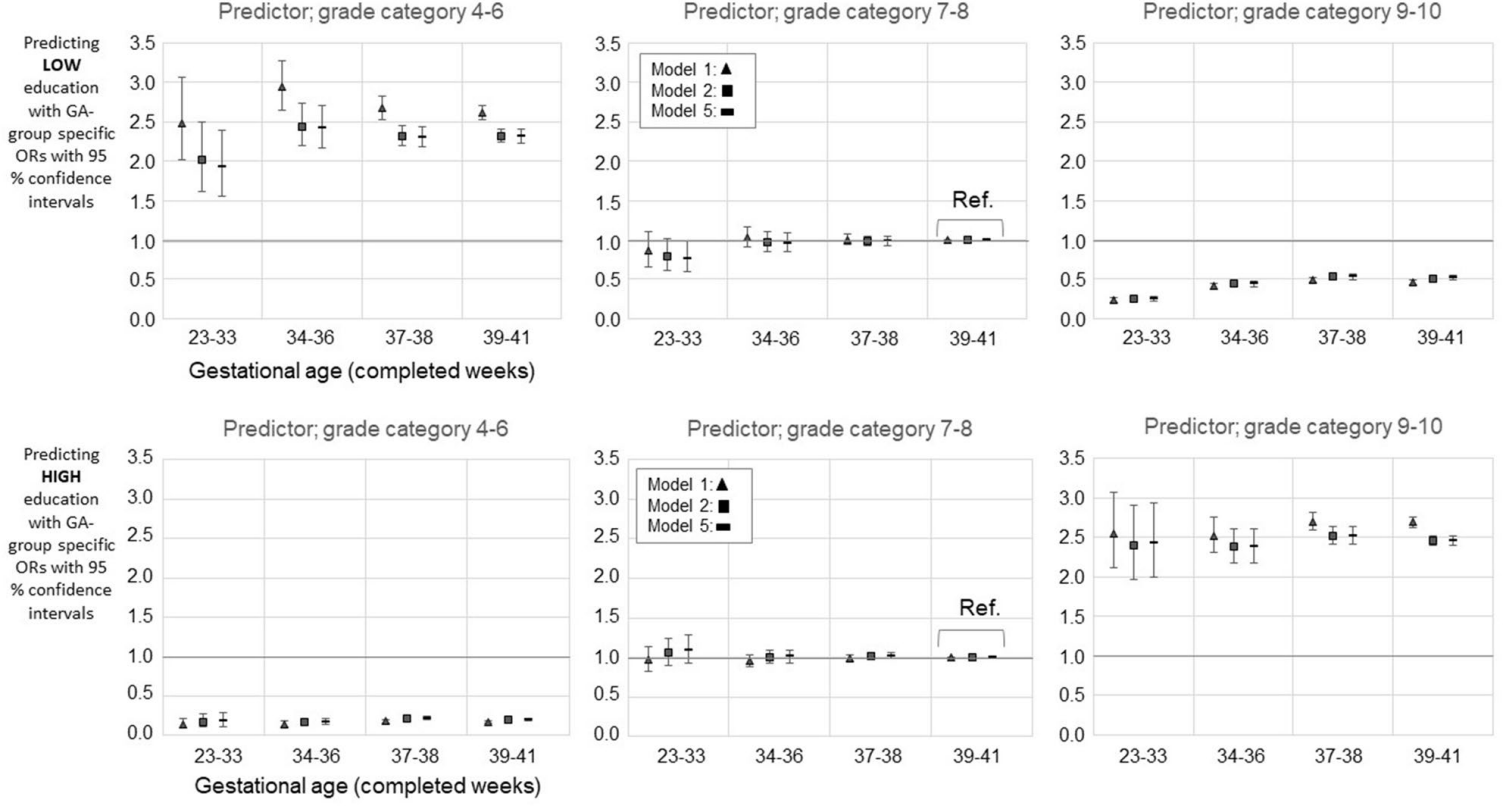
Mathematics grade and gestational age category together predicting [LOW] ‘basic only or unknown’ (upper panel) and [HIGH] ‘lower tertiary or more’ (lower panel) education. Comparisons to intermediate education i.e., ‘upper secondary, less than tertiary’. GA 39–41 and grade category 7–8 serves as a reference group. The figures show models 1, 4, and 5. Models 2 and 3 are available in the Supplementary Table 10ab. The *p* values (for Model 5) from the comparisons of interaction- and main-effects-only models were 0.721 for grade 4–6 group; 0.638 for grade 7–8 group; and 0.718 for grade 9–10 group. For unadjusted model (Model 1) the *p* values from the comparisons were as follows: 0.599 for grade 4–6 group; 0.785 for grade 7–8 group; and 0.704 for grade 9–10 group. Only such individuals who attended mainstream education in compulsory education are included. Model 1; Unadjusted model, Model 4; Adjusted for the sex, birth year, maternal and paternal ages, maternal and paternal highest attained education, BWSDS, gestational disorder(s), maternal smoking at pregnancy, maternal marital status at the childbirth, and birth order, Model 5; Adjusted as Model 4, and for severe medical condition.

