# Supplementary material for: School grades and educational attainments of adolescents and young adults born preterm
Source: Sci Rep. 2023 Jan 5;13:231. doi: 10.1038/s41598-022-27295-4 (PMC9816170; doi:10.1038/s41598-022-27295-4)
Supplement: Supplementary file 1 — Supplementary Information. [file 41598_2022_27295_MOESM1_ESM.docx]

# SUPPLEMENTARY INFORMATION

Alenius, S., Kajantie, E., Sund, R., Nurhonen, M., Haaramo, P., Näsänen-Gilmore P., Lemola, S., Räikkönen, K., Schnitzlein, D.D., Wolke, D., Gissler, M., Hovi, P.

School grades and educational attainments of adolescents and young adults born preterm

1. Supplementary methods
   1. Description of the registers
      1. Statistics Finland
      2. Social Insurance Institution (SII)
   2. Finnish education system
      1. Special education
   3. Supplementary analyses
   4. Supplementary references
2. Supplementary tables

**Table S1:** Diagnosis codes included in the covariates on severe medical condition following Moster et al.

**Table S2**: Univariate Association (ORs and 95% CIs) between individual covariates and educational level at 25 years of age for those who attended mainstream education.

**Table S3**: Number of individuals in special education according to GA category, and Odds Ratios (OR) with 95% Confidence Intervals for attending special education in compulsory education.

**Table S4**: Number of individuals discontinuing compulsory education according to GA category, and Odds Ratios (OR) with 95% Confidence Intervals for discontinuing compulsory education.

**Table S5**: The number of individuals with a mathematics grade in mainstream education, and the variation in mathematics grades at 16 yr of age according to GA category in mainstream education.

**Table S6**: The number of individuals with a grade on theoretical subjects in mainstream education, and the variation in grades on theoretical subjects at 16 yr of age according to GA category in mainstream education.

**Table S7**: The number of individuals with a native language grade in mainstream education, and the variation in grades on native language at 16 yr of age according to GA category in mainstream education.

**Table S8:** The number of individuals with a physical education grade in mainstream education, and the variation in grades in physical education at 16 yr of age according to GA category in mainstream education.

**Table S9ab**: Number of individuals with low education (basic only or unknown education) / high education (lower tertiary or higher education) at 25 years of age according to GA category, and Odds Ratios (OR) with 95% Confidence Intervals for basic only or unknown education.

**Table S10ab**: Odds Ratios and 95% Confidence Intervals for low education (basic or unknown education) / high education (lower tertiary or more) at 25 years of age according to gestational age category and grade category in mathematics at 16 years of age as compared to secondary, less than tertiary education.

**Table S11ab:** Odds Ratios and 95% Confidence Intervals for low education (basic or unknown education) / high education (lower tertiary or more) at 25 years of age according to gestational age category and grade category in theoretical subjects at 16 years of age as compared to secondary, less than tertiary education.

**Table S12ab**: Odds Ratios and 95% Confidence Intervals for low education (basic or unknown education) / high education (lower tertiary or more) at 25 years of age according to gestational age category and grade category in native language at 16 years of age as compared to secondary, less than tertiary education.

**Table S13ab:** Odds Ratios and 95% Confidence Intervals for low education (basic or unknown education) / high education (lower tertiary or more) at 25 years of age according to gestational age category and grade category in physical education at 16 years of age as compared to secondary, less than tertiary education.

1. Supplementary Figures

**Figure S1**: Odds Ratios (ORs) and 95% Confidence Intervals (CIs) for discontinuing education in compulsory education according to gestational age category.

**Figure S2**: The mean of theoretical subjects and gestational age category together predicting [LOW] ‘basic only or unknown’ (upper panel) and [HIGH] ‘lower tertiary or more’ (lower panel) education. Comparisons to intermediate education i.e., ‘upper secondary, less than tertiary’.

**Figure S3**: Native language grade and gestational age category together predicting [LOW] ‘basic only or unknown’ (upper panel) and [HIGH] ‘lower tertiary or more’ (lower panel) education. Comparisons to intermediate education i.e., ‘upper secondary, less than tertiary’.

**Figure S4**: Physical education (PE) grade and gestational age category together predicting [LOW] ‘basic only or unknown’ (upper panel) and [HIGH] ‘lower tertiary or more’ (lower panel) education. Comparisons to intermediate education i.e., ‘upper secondary, less than tertiary’.

## SUPPLEMENTARY METHODS

### 1.1 Description of the Registers

#### 1.1.1 Statistics Finland

**Statistics Finland** (SF) is a public authority in Finland. It combines collected data and produces most Finnish official statistics. The SF maintains amongst other register data individual level information on educational attainments at the end of the compulsory school. Data on educational attainments is based on the information originating from the National Joint Application Register maintained by the National Board of Education. The data provided for this study by SF include information on school grades in the school leaving diploma at the end of the compulsory school (in general at 16 years of age), the form of the education received (whether the study subject attended mainstream education or special education), and the year of achieving the diploma.

The data on school grades cover 19 school subjects (native language, foreign languages, religion, history, mathematics, physics, chemistry, biology, geography, physical education, music, arts, handcraft, home economics, philosophy, and psychology) along with the mean grade of all of the theoretical subjects combined namely the mean grade of native language, foreign languages, religion, history, mathematics, physics, chemistry, biology, geography, philosophy, and psychology. We had information on grades and on the type of the education received for every individual who participated in the national joint application procedure of upper secondary education within years 2003 to 2007.

Statistics Finland also provides data on achieved education levels. We had data on achieved educational levels through 2015. The information on achieved education in Statistics Finland’s data was divided in five categories; I) upper secondary (corresponding International Standard Classification of Education (ISCED) 3), II) post-secondary, non-tertiary (ISCED 5), III) lower tertiary (ISCED level 6), IV) upper tertiary (ISCED level 7) and V) doctoral (ISCED level 8).[1] Statistics Finland do not register ISCED levels of 0 to 2 (early childhood education, primary education, lower secondary education) at an individual level. Thus, we computed an additional sixth category VI) basic only or unknown for those individuals (and their parents) who did not have data on education.

#### 1.1.2 Social Insurance Institution (SII)

**The Social Insurance Institution (SII)** is an independent social security institution supervised by the Finnish Parliament. To its responsibilities include basic social security system financed by tax. SII also provides a range of benefits such as maternity grant, student financial aid for post-comprehensive school education, and disability allowances. Hence, the SII maintains a variety of nationwide individual level detailed information on which the issuing of each benefit is based on.

Disability allowance is granted in order to provide financial support in daily life when a person has a disability or chronic illness. The allowance is based on a medical statement prepared by a physician. The statement includes information on the disability (diagnosis code and name), the number of regular visits to the physician, information on whether a child younger than 16 years of age, because of the disability, needs more supervision and help in everyday life than his/her peers without any disabling condition. It also encompasses information on whether the child has special needs at home, in day care, or at school, as well as if the child receives in rehabilitation.[2]

### 1.2 The Finnish education system

The Finnish education system constitutes of a 9-year compulsory school for children most often between 7 and 16 years of age. On some occasions it is possible to complete compulsory education later than at 16 years of age. That may indicate one year later school start due to non-school- readiness according to psychological test, two years long preschool among those severely disabled who attend prolonged CE, repetition of a school year, or attending to voluntary additional basic education.[3] All Finnish schools employ a national core syllabus, which includes objectives and core contents of different subjects. After finishing compulsory school, pupils receive a basic education certificate, providing assessments on all school subjects, each with a score from 4 fail, 5 adequate, 6 moderate, 7 satisfactory, 8 good, 9 very good, to 10 excellent skills. The certificate serves as a selection criterion when applying to post-compulsory education.

Post-compulsory education comprises of (1) general or (2) vocational upper secondary education, of which the three-year general secondary education leads to matriculation examination and enable further studies at a higher education institution i.e., studies at universities (with emphasis on scientific research) or at universities of applied sciences (emphasis on more practical approach to the science). The three-year vocational upper secondary education provides vocational qualification, which most often combined to work experience, can permit certain studies at a higher education institution. No tuition fees for university studies exist in Finland. As there are more applicants to the universities and to universities of applied sciences than there are annual vacant places at these institutions, the institutions therefore employ a variety of student selection criteria, such as success in matriculation examination, entrance tests, and work experience or the combination there of.[3]

#### 1.2.1 Special education

In Finland, the education in compulsory school is divided in mainstream education, and to education that include varying amount of extra support. Those who need other than general extra support (i.e., support entitled for everyone as a part of the everyday teaching including short term individual or small-group supportive/remedial teaching) and have special grounds (please, see below) may be accepted to special education. Decisions on special education are official decisions that are preceded by multidisciplinary evaluation and discussions within school authorities and with the guardian(s) of the pupil. A pupil may attend special education in one or more, or in all school subjects. Decisions on special education are recorded in the administrative registers (and are therefore available for example for research purposes as is case in this study). In our study we lacked data on in which school subjects a pupil attended special education, therefore a pupil was, in our work, considered to have attended mainstream education if he/she had a diploma indicating no participation in special education in any of the school subjects.

In 2001-2010 the grounds for special education were (1) slightly to severely delayed development, (2) varying degrees of cerebral dysfunction, physical disability or similar (including ADHD and cerebral palsy for example), (3) emotional disturbance or social maladjustment, (4) learning difficulties related to autism or Asperger’s syndrome, (5) learning difficulties caused by dysphasia, (6) visual or hearing impairments, and (7) miscellaneous grounds.[4] The principles of the Finnish special education grouping largely follow those of employed in the US[5,6] and other European Union countries.[7]

Students who have attended special education are eligible to continue to upper secondary education and may apply to ordinary vocational institutions within the national joint application system for further education or through the related flexible application procedure.[8]

### 1.3 Supplementary analyses

As showed in the Results section of the main manuscript, the gestational age of the individual was not associated to discontinuing compulsory education in models not adjusted with any of the covariates. Supplementary univariate analyses indicated that *severe medical condition* (odds ratio [OR] 1.57, 95% confidence interval [CI]1.15 - 2.13) and the *birth years of 1989 and 1990* (ORs 0.24 (CI 0.19 - 0.32), 0.21 (CI 0.16 - 0.29) respectively) of the individual were associated to discontinuing compulsory education. Further, *maternal and paternal ages less than 20yr* (ORs 2.65 (CI 1.96 - 3.58), 3.15 (CI 1.81 - 5.48) respectively), and *maternal and paternal low education* (ORs 2.61 (CI 2.20 - 3.10), 1.65 (1.39 - 1.96) respectively) were risk factors for discontinued education in compulsory education, whereas *maternal and paternal high educational levels* were associated to lower likelihood for discontinuing compulsory education; ORs 0.48 (CI 0.36 - 0.64) and 0.44 (CI 0.33 - 0.60). Maternal *smoking in pregnancy* (OR 3.01 (CI 2.55 - 3.55)), and mother being *unmarried* (OR 1.92 (CI 1.62 - 2.26)) served as risk factor for discontinuing compulsory education. The sex of the individual, smallness for the GA (SGA), birth order, birth year of 1988 (as compared to 1987) and multiple birth were unassociated to discontinuing compulsory education, as were maternal gestational disorders and parental age(s) over 34 years (as compared to parental age of 20 to 34 years).

### 1.4 Supplementary references

1. Official Statistics of Finland. Classifications; The level of education (Finnish National Agency for Education): Classification of levels of education used in the Educational Administration 2002. https://www2.stat.fi/en/luokitukset/koulutusaste_oh/koulutusaste_oh_1_20050101/. Accessed 10 Oct 2021. No page update history available.

Social Insurance Institution (SII). Description of statistics; Statistics on disability benefits provided by Kela. Available at: https://www.kela.fi/en_US/web/en/description-of-statistics3. Accessed 10 Oct 2021. No page update history available.

1. Finnish National Agency for Education: Finnish education system [Webpage]. Available at: http://www.oph.fi/en/education-system. Accessed 2 Dec 2020. No page update history available.
2. Official Statistics of Finland (OSF): Support for learning [e-publication]. ISSN=1799-1617. Helsinki: Statistics Finland [referred: 26.5.2022]. Access method: http://www.stat.fi/til/erop/kas_en.html
3. Avchen, R.N., Scott, K.G. & Mason, C.A. Birth weight and school-age disabilities: a population-based study. *Am. J. Epidemiol*. **154,** 895-901 (2001).
4. Msall, M.E., Avery, R.C., Tremant, M.R., Lima, J.C., Rogers, M.L. & Hogan, D.P. Functional disability and school activity limitations in 41,300 school-age children: relationship to medical impairments. *Pediatrics*. **111,** 548-553 (2003).
5. European agency for special needs and inclusive education. Editor: Meijer CJW. Special education across Europe in 2003. Available at: http://www.european-agency.org /resources/publications/special-education-across-europe-2003. Accessed 26 Nov 2020. Page last updated: 18 Mar 2020.
6. European Commission; EACEA National Policies Platform: Eurydice. Finland: Special educational needs provision within mainstream education. Accessed 10 Oct 2021. Page Last Updated 10 Oct 2021.

## SUPPLEMENTARY TABLES

### Supplementary Table S1.

| **Supplementary Table S1**. Diagnosis codes included in the covariates on severe medical condition following Moster et al.^19^ | | |
| --- | --- | --- |
| *Medical disability^a^* | ICD-9 | ICD-10 |
| Cerebral palsy | 342-344 | G80-83 |
| Mental retardation | 317-319 | F70-F79 |
| Schizophrenia | 295 | F20-21 |
| Autism spectrum | 299 | F84 |
| Disorders of psychological development, behavior and emotion | 3070, 3072-3073, 3075-3077, 3079, 3092, 3098, 312-315, 7846 | F80-83, F88-F98 |
| *Other major disabilities^a^* | | ICD-10 |
| Epilepsy | 345 | G40-41 |
| Blindness, low vision | 369 | H54 |
| Hearing loss | 389 | H90-91 |

a The data on medical disabilities and other major disabilities are derived from the register on disability allowance maintained by the Social Insurance Institution (SII). An individual was considered to have medical condition and/or other major disability if any of the diagnosis codes presented appeared in the register on disability allowances before 16 years of age on 31st December 2015.

ICD-9 – International Classification of Diseases, ninth edition

ICD-10 – International Classification of Diseases and Related Health Problems, tenth edition.

### Supplementary Table S2.

| **Supplementary Table S2.** Univariate Association (ORs and 95% CIs) between individual covariates and educational level at 25 years of age for those who attended mainstream education; Basic only or unknown (low education), or Lower tertiary or more (high education) as compared to Upper secondary or nontertiary (intermediate education). | | |
| --- | --- | --- |
|  | **Basic education only or unknown** | **Lower tertiary or more** |
| Independent effect of covariates | OR (95% CI) | OR (95% CI) |
| Sex (*male* vs. *female*) | 1.10 (1.07 - 1.13) | 0.42 (0.41 - 0.43) |
| Small for gestational age (SGA) (*yes* vs. *no*) | 1.35 (1.24 -1.47) | 0.82 (0.77 - 0.88) |
| Severe medical condition (*yes* vs. *no*) ^a^ | 1.63 (1.53 -1.75) | 0.51 (0.47 - 0.55) |
| First born (*yes* vs. *no*) ^b^ | 0.99 (0.96 - 1.02)*^P^* ^=.361^ | 1.15 (1.13 - 1.18) |
| Multiple birth (*yes* vs. *no*) | 0.91 (0.82 - 1.01)*^P^* ^=.084^ | 1.07 (1.00-1.14)*^P^*^=.068^ |
| Birth year (*1987* vs. *1988* or *1989* or *1990*) | | |
| 1988 | 0.98 (0.94 - 1.02)*^P^* ^=.272^ | 1.03 (1.00 - 1.06)*^P^* ^=.027^ |
| 1989 | 0.97 (0.93 - 1.01)*^P^* ^=.084^ | 1.02 (0.99 - 1.05)*^P^* ^=.161^ |
| 1990 | 0.98 (0.94 - 1.02)*^P^* ^=.380^ | 1.04 (1.01 - 1.07)*^P^* ^=.005^ |
| Maternal age *20-34yr* *(incl. Missing data)* vs. *less than 20yr* or *35yr or more* | | |
| Less than 20yr | 2.38 (2.24 - 2.54) | 0.35 (0.32 - 0.39) |
| 35yr or more | 0.83 (0.79 - 0.87) | 1.15 (1.12 - 1.19) |
| Unknown maternal age | na. | na. |
| Maternal marital status (*married* vs. *unmarried* or *unknown marital status)* ^c^ | | |
| Unmarried | 1.77 (1.72 - 1.83) | 0.60 (0.58 - 0.62) |
| Unknown marital status | 1.74 (1.45 - 2.09) | 0.78 (0.66 - 0.92)*^P^* ^=.003^ |
| Maternal smoking in pregnancy (*non-smoker* vs. *smoker* or *unknown smoking status*) | | |
| Smoker | 2.24 (2.17 - 2.32) | 0.47 (0.45 - 0.48) |
| Unknown smoking status | 1.25 (1.12 - 1.38) | 0.85 (0.79 - 0.92) |
| Maternal gestational disorder (*yes* vs. *no*) ^d^ | 0.95 (0.91 - 1.00)*^P^* ^=.043^ | 1.02 (0.99 - 1.05)*^P^* ^=.289^ |
| Maternal highest attained education (*Intermediate* vs. *Low (incl. Missing data),* or *High*) | | |
| Low | 2.03 (1.96 - 2.11) | 0.55 (0.53 - 0.57) |
| High | 0.65 (0.62 - 0.68) | 1.75 (1.71 - 1.79) |
| Maternal education data missing | na. | na. |
| Paternal age *20-34yr* vs. *less than 20yr* or *35yr or more* | | |
| Less than 20yr | 2.59 (2.28 - 2.93) | 0.33 (0.27 - 0.40) |
| 35yr or more | 0.84 (0.81 - 0.87) | 1.08 (1.06 - 1.11) |
| Unknown paternal age | 2.19 (1.98 - 2.43) | 0.51 (0.45 - 0.58) |
| Paternal highest attained education (*Intermediate* vs. *Low*, or *High*, or *Data missing*) | | |
| Low | 1.57 (1.52 - 1.62) | 0.70 (0.68 - 0.72) |
| High | 0.59 (0.56 - 0.62) | 1.87 (1.38 - 1.92) |
| Paternal education data missing | 2.38 (2.15 - 2.64) | 0.55 (0.48 - 0.62) |
| All *P*-values were <.001 if not otherwise denoted  na. - not applicable | |  |
| a Severe medical condition - received disability allowance before 16 years of age due to severe medical condition. | | |
| b First born - the individual is born as a first child to his/her mother. Adoptive siblings are included. | | |
| c Maternal marital status at the birth of the individual. | | |
| d Gestational disorder includes gestational diabetes, gestational hypertensive disorder, and intrahepatic cholestasis of pregnancy. | | |
| Multiple birth was not adjusted for in the analyses. | | |
|  |  |  |

### Supplementary Table S3.

| **Supplementary Table S3.** Number of individuals in **special education** according to GA category, and Odds Ratios (OR) with 95% Confidence Intervals for attending special education in compulsory education. | | | | | | | | |
| --- | --- | --- | --- | --- | --- | --- | --- | --- |
| GA^a^ | 23-27 weeks | 28-31 weeks | 32-33 weeks | 34-36 weeks | 37-38 weeks | 39-41 weeks | 42 weeks | Total |
| n | 240 | 844 | 1,149 | 8,288 | 39,751 | 164,474 | 8,988 | 223,744 |
| Special education, n (%) | 48 (20.0) | 110 (13.0) | 87 (7.6) | 540 (6.5) | 2,088 (5.3) | 7,575 (4.6) | 433 (4.8) | 10,881 (4.9) |
|  | OR (95% CI) | OR (95% CI) | OR (95% CI) | OR (95% CI) | OR (95% CI) | OR (95% CI) | OR (95% CI) | . |
| 1 | 5.48 (3.98-7.55) | 3.19 (2.61-3.91) | 1.73 (1.39-2.15) | 1.45 (1.33-1.59) | 1.15 (1.09-1.21) | Ref. | 1.05 (0.95-1.16)*^P^* ^=.316^ | . |
| 2 | 5.54 (4.01-7.65) | 3.00 (2.45-3.69) | 1.65 (1.32-2.05) | 1.40 (1.28-1.54) | 1.13 (1.08-1.19) | Ref. | 1.03 (0.93-1.14)*^P^* ^=.597^ | . |
| 3 | 5.36 (3.84-7.49) | 2.94 (2.38-3.62) | 1.52 (1.21-1.90) | 1.32 (1.20-1.45) | 1.10 (1.05-1.16) | Ref. | 1.02 (0.92-1.12)*^P^* ^=.772^ | . |
| 4 | 5.43 (3.87-7.60) | 2.90 (2.35-3.58) | 1.47 (1.17-1.84) | 1.29 (1.17-1.41) | 1.09 (1.03-1.15) | Ref. | 1.04 (0.94-1.16)*^P^* ^=.406^ | . |
| 5 | 2.19 (1.52-3.16) | 1.67 (1.33-2.10) | 1.13 (0.89-1.44)*^P^* ^=.314^ | 1.20 (1.09-1.32) | 1.07 (1.01-1.12)*^P^* ^=.019^ | Ref. | 1.01 (0.91-1.12)*^P^* ^=.819^ | . |
| a GA - gestational age (completed weeks) | | | |  |  |  |  |  |
| The *P*-value is less than .001 if not otherwise denoted. | | | | |  |  |  |  |
| Model 1: Unadjusted Model | | |  |  |  |  |  |  |
| Model 2: Adjusted for the sex, birth year, and for maternal and paternal ages at the birth of the individual | | | | | | | | |
| Model 3: Adjusted as Model 2 + maternal and paternal highest attained education | | | | | | |  |  |
| Model 4: Adjusted as Model 3 + BWSDS, gestational disorder(s), maternal smoking at pregnancy, maternal marital status at the birth of the individual, and birth order | | | | | | | | |
| Model 5: Adjusted as Model 4 + severe medical condition of the individual | | | | | | |  |  |

### Supplementary Table S4.

| **Supplementary Table S4.** Number of individuals **discontinuing compulsory education** according to GA category, and Odds Ratios (OR) with 95% Confidence Intervals for discontinuing compulsory education. | | | | | | | | |
| --- | --- | --- | --- | --- | --- | --- | --- | --- |
| GA^a^ | 23-27 weeks | 28-31 weeks | 32-33 weeks | 34-36 weeks | 37-38 weeks | 39-41 weeks | 42 weeks | Total |
| n | 240 | 844 | 1,149 | 8,288 | 39,751 | 164,474 | 8,988 | 223,744 |
| Discontinued education, n (%) | 7 (0.3) ^b^ | | | 15 (0.2) | 129 (0.3) | 450 (0.3) | 26 (0.3) | 627 (0.3) |
|  | OR (95% CI) | OR (95% CI) | OR (95% CI) | OR (95% CI) | OR (95% CI) | OR (95% CI) | OR (95% CI) | . |
| 1 | 3.85 (0.95-15.54)*^P^* ^= .059^ | 1.46 (0.47-4.57)*^P^* ^=.511^ | 0.67 (0.17-2.68)*^P^* ^=.570^ | 0.68 (0.41-1.14)*^P^* ^=.141^ | 1.20 (0.98-1.46)*^P^* ^=.073^ | Ref. | 1.06 (0.72-1.58)*^P^* ^=.761^ | . |
| 2 | 3.68 (0.91-14.95)*^P^* ^=.068^ | 1.42 (0.46-4.45)*^P^* ^=.545^ | 0.64 (0.16-2.58)*^P^* ^=.533^ | 0.66 (0.39-1.10)*^P^* ^=.107^ | 1.18 (0.97-1.44)*^P^* ^=.092^ | Ref. | 1.08 (0.73-1.61)*^P^* ^=.069^ | . |
| 3 | 3.54 (0.87-14.42)*^P^* ^=.078^ | 1.42 (0.46-4.46)*^P^* ^=.543^ | 0.58 (0.14-2.34)*^P^* ^=.445^ | 0.62 (0.37-1.04)*^P^* ^=.067^ | 1.15 (0.95-1.40)*^P^* ^=.155^ | Ref. | 1.06 (0.71-1.58)*^P^* ^=.771^ | . |
| 4 | 3.42 (0.84-14.00)*^P^* ^=.087^ | 1.38 (0.44-4.31)*^P^* ^=.384^ | 0.56 (0.14-2.24)*^P^* ^=.410^ | 0.59 (0.35-1.00)*^P^* ^=.048^ | 1.13 (0.93-1.38)*^P^* ^=.232^ | Ref. | 1.08 (0.72-1.60)*^P^* ^=.712^ | . |
| 5 | 2.62 (0.64-10.76)*^P^* ^=.183^ | 1.20 (0.38-3.77)*^P^* ^=.756^ | 0.53 (0.13-2.12)*^P^* ^=.367^ | 0.59 (0.35-0.98)*^P^* ^=.043^ | 1.12 (0.92-1.37)*^P^* ^=.247^ | Ref. | 1.07 (0.72-1.59)*^P^* ^=.737^ | . |
| a GA - gestational age (completed weeks) | | | |  |  |  |  |  |
| b Gestational age categories of 23 to 33 weeks are combined because privacy regulations prevent us to display cell counts of three or less. | | | | | | | | |
| The *P*-value is less than .001 if not otherwise denoted. | | | | |  |  |  |  |
| Model 1: Unadjusted Model | | |  |  |  |  |  |  |
| Model 2: Adjusted for the sex, birth year, and for maternal and paternal ages at the birth of the individual | | | | | | | | |
| Model 3: Adjusted as Model 2 + maternal and paternal highest attained education | | | | | | |  |  |
| Model 4: Adjusted as Model 3 + BWSDS, gestational disorder(s), maternal smoking at pregnancy, maternal marital status at the birth of the individual, and birth order | | | | | | | | |
| Model 5: Adjusted as Model 4 + severe medical condition of the individual | | | | | | |  |  |

### Supplementary Table S5.

| **Supplementary Table S5.** The number of individuals ^a^ with a mathematics grade in mainstream education, and the variation in mathematics grades at 16 yr of age according to GA category in mainstream education expressed as grade differences and Z-scores with 95% Confidence Intervals | | | | | | | | | | | | | | |
| --- | --- | --- | --- | --- | --- | --- | --- | --- | --- | --- | --- | --- | --- | --- |
| GA^b^ | | 23-27 weeks | 28-31 weeks | 32-33 weeks | | 34-36 weeks | 37-38 weeks | | 39-41 weeks | | 42 weeks | | 23-42 weeks | |
| n (%) ^a^ | | 174 (97.8) | 696 (99.3) | 1,017 (99.3) | | 7,531 (99.7) | 36,736 (99.5) | | 153,306 (99.6) | | 8,320 (99.5) | | 207,780 (99.6) | |
| **Grade differences** | | | | | | | | | | | | | | |
| Grade, mean (SD) | | 7.22 (1.31) | 7.37 (1.35) | 7.52 (1.34) | | 7.52 (1.38) | 7.54 (1.38) | | 7.54 (1.37) | | 7.51 (1.37) | | 7.54 (1.37) | |
| Model 1 | | -0.33 (-0.53 to -0.12)*^P^* ^=.002^ | -0.18 (-0.28 to -0.07)*^P^* ^=.001^ | -0.02 (-0.11 to 0.06)*^P^* ^=.604^ | | -0.02 (-0.05 to 0.01)*^P^* ^=.230^ | -0.00 (-0.02 to 0.02)^P =.898^ | | Ref. | | -0.03 (-0.06 to 0.00)*^P^* ^=.072^ | | . | |
| Model 2 | | -0.33 (-0.54 to -0.13)*^P^* ^=.001^ | -0.15 (-0.25 to -0.05)*^P^* ^=.004^ | -0.00 (-0.09 to 0.08)*^P^* ^=.936^ | | -0.01 (-0.04 to 0.03)*^P^* ^=.693^ | 0.00 (-0.01 to 0.02)*^P^* ^=.710^ | | Ref. | | -0.01 (-0.04 to 0.02)*^P^* ^=.352^ | | . | |
| Model 3 | | -0.27 (-0.46 to -0.08)*^P^* ^=.006^ | -0.12 (-0.22 to -0.03)*^P^* ^=.013^ | 0.04 (-0.04 to 0.12)*^P^* ^=.364^ | | 0.03 (-0.00 to 0.06)*^P^* ^=.063^ | 0.01 (0.00 to 0.03)*^P^* ^=.059^ | | Ref. | | -0.01 (-0.04 to 0.02)*^P^* ^=.605^ | | . | |
| Model 4 | | -0.27 (-0.46 to -0.08)*^P^* ^=.006^ | -0.13 (-0.22 to -0.03)*^P^* ^=.010^ | 0.04 (-0.04 to 0.12)*^P^* ^=.298^ | | 0.03 (-0.00 to 0.06)*^P^* ^=.064^ | 0.02 (0.00 to 0.03)*^P^* ^=.019^ | | Ref. | | -0.03 (-0.06 to 0.00)*^P^* ^=.058^ | | . | |
| Model 5 | | -0.19 (-0.38 to -0.00)*^P^* ^=.046^ | -0.09 (-0.19 to 0.01)*^P^* ^=.065^ | 0.05 (-0.03 to 0.13)*^P^* ^=.190^ | | 0.03 (0.00 to 0.06)*^P^* ^=.036^ | 0.02 (0.00 to 0.03)*^P^* ^=.013^ | | Ref. | | -0.03 (-0.06 to 0.00)*^P^* ^=.068^ | | . | |
| **Z-scores** | | | | | | | | | | | | | | |
| Z-score, mean (SD) | | -0.23 (0.95) | -0.13 (0.98) | -0.01 (0.97) | | -0.01 (1.00) | 0.00 (1.00) | | 0.00 (1.00) | | -0.02 (0.99) | | 0.00 (1.00) | |
| Model 1 | | -0.24 (-0.39 to -0.09)*^P^* ^=.002^ | -0.13 (-0.20 to -0.05)*^P^* ^=.001^ | -0.02 (-0.08 to 0.05)*^P^* ^=.604^ | | -0.01 (-0.04 to 0.09)*^P^* ^=.230^ | -0.00 (-0.01 to 0.01)*^P^* ^=. 898^ | | Ref. | | -0.02 (-0.04 to 0.00)*^P^* ^=.072^ | | . | |
| Model 2 | | -0.24 (-0.39 to -0.09)*^P^* ^=.001^ | -0.11 (-0.18 to -0.04)*^P^* ^=.004^ | -0.00 (-0.06 to 0.06)*^P^* ^=.936^ | | -0.01 (-0.03 to 0.02)*^P^* ^=.693^ | 0.00 (-0.01 to 0.01)*^P^* ^=.710^ | | Ref. | | -0.01 (-0.03 to 0.01)*^P^* ^=.352^ | | . | |
| Model 3 | | -0.20 (-0.34 to -0.06)*^P^* ^=.006^ | -0.09 (-0.16 to -0.02)*^P^* ^=.013^ | 0.03 (-0.03 to 0.09)*^P^* ^=.364^ | | 0.02 (-0.00 to 0.04)*^P^* ^=.063^ | 0.01 (0.00 to 0.02)*^P^* ^=.059^ | | Ref. | | -0.01 (-0.03 to 0.02)*^P^* ^=.605^ | | . | |
| Model 4 | | -0.19 (-0.33 to -0.06)*^P^* ^=.006^ | -0.09 (-0.16 to -0.02)*^P^* ^=.010^ | 0.03 (-0.03 to 0.09)*^P^* ^=.298^ | | 0.02 (0.00 to 0.04)*^P^* ^=.064^ | 0.01 (0.00 to 0.02)*^P^* ^=.019^ | | Ref. | | -0.02 (-0.04 to 0.00)*^P^* ^=.058^ | | . | |
| Model 5 | | -0.14 (-0.28 to -0.00)*^P^* ^=.046^ | -0.07 (-0.14 to 0.00)*^P^* ^=.065^ | 0.04 (-0.02 to 0.10)*^P^* ^=.190^ | | 0.02 (0.00 to 0.05)*^P^* ^=.036^ | 0.01 (0.00 to 0.02)*^P^* ^=.013^ | | Ref. | | -0.02 (-0.04 to 0.00)*^P^* ^=.068^ | | . | |
| b GA - gestational age (completed weeks) | | | | |  | |  | |  | |  |  |  |  |
|  | | | | | | | | | | | | |  |  |
| The *P*-value is less than .001 if not otherwise denoted. | | | | | | |  | |  | |  |  |  |  |
| Model 1: Unadjusted Model | | | |  |  | |  | |  | |  |  |  |  |
| Model 2: Adjusted for the sex, birth year, and for maternal and paternal ages at the birth of the individual | | | | | | | | | | | | |  |  |
| Model 3: Adjusted as Model 2 + maternal and paternal highest attained education | | | | | | | | | | |  |  |  |  |
| Model 4: Adjusted as Model 3 + BWSDS, gestational disorder(s), maternal smoking at pregnancy, maternal marital status at the birth of the individual, and birth order | | | | | | | | | | | | |  |  |
| Model 5: Adjusted as Model 4 + severe medical condition of the individual | | | | | | | | | | |  |  |  |  |

### Supplementary Table S6.

| **Supplementary Table S6.** The number of individuals ^a^ with a grade on theoretical subjects in mainstream education, and the variation in grades on theoretical subjects at 16 yr of age according to GA category in mainstream education expressed as grade differences and as z-scores with 95% confidence intervals | | | | | | | | |
| --- | --- | --- | --- | --- | --- | --- | --- | --- |
| GA^b^ | 23-27 weeks | 28-31 weeks | 32-33 weeks | 34-36 weeks | 37-38 weeks | 39-41 weeks | 42 weeks | 23-42 weeks |
| n (%) ^a^ | 172 (96.6) | 701 (99.3) | 1,015 (99.1) | 7,518 (99.5) | 36,685 (99.4) | 153,055 (99.4) | 8,296 (99.2) | 207,437 (99.4) |
| **Grade differences** | | | | | | | | |
| Grade, mean (SD) | 7.66 (1.08) | 7.68 (1.11) | 7.73 (1.08) | 7.68 (1.09) | 7.68 (1.10) | 7.68 (1.10) | 7.66 (1.08) | 7.68 (1.09) |
| 1 | -0.02 (-0.19 to 0.14)*^P^* ^= .781^ | -0.01 (-0.09 to 0.08)*^P^* ^=.905^ | 0.05 (-0.02 to 0.12)*^P^* ^=.138^ | -0.00 (-0.03 to 0.02)*^P^* ^=.845^ | 0.00 (-0.01 to 0.01)*^P^* ^=.998^ | Ref. | -0.02 (-0.05 to 0.00)*^P^* ^=.084^ | . |
| 2 | -0.03 (-0.18 to 0.13)*^P^* ^=.741^ | 0.03 (-0.04 to 0.11)*^P^* ^=.387^ | 0.08 (0.01 to 0.14)*^P^* ^=.021^ | 0.02 (-0.08 to 0.04)*^P^* ^=.194^ | 0.01 (-0.00 to 0.02)*^P^* ^=.182^ | Ref. | -0.01 (-0.03 to 0.02)*^P^* ^=.690^ | . |
| 3 | 0.03 (-0.12 to 0.17)*^P^* ^=.708^ | 0.06 (-0.01 to 0.13)*^P^* ^=.109^ | 0.11 (0.05 to 0.17) | 0.05 (0.02 to 0.07) | 0.02 (0.01 to 0.03)*^P^* ^=.001^ | Ref. | 0.00 (-0.02 to 0.02)*^P^* ^=.915^ | . |
| 4 | 0.03 (-0.11 to 0.17)*^P^* ^=.678^ | 0.04 (-0.03 to 0.11)*^P^* ^=.262^ | 0.10 (0.04 to 0.16)*^P^* ^=.001^ | 0.04 (0.02 to 0.06)*^P^* ^=.001^ | 0.02 (0.01 to 0.03)*^P^* ^=.001^ | Ref. | -0.02 (-0.05 to -0.00)*^P^* ^=.028^ | . |
| 5 | 0.09 (-0.05 to 0.24)*^P^* ^=.201^ | 0.07 (0.00 to 0.11)*^P^* ^=.047^ | 0.11 (0.05 to 0.17) | 0.04 (0.02 to 0.06) | 0.02 (0.01 to 0.03) | Ref. | -0.02 (-0.04 to -0.00)*^P^* ^=.033^ | . |
| **Z-scores** | | | | | | | | |
| Z-score, mean (SD) | -0.02 (0.99) | 0.00 (1.01) | 0.05 (0.99) | 0.00 (1.00) | 0.00 (1.01) | 0.00 (1.00) | -0.02 (0.99) | 0.00 (1.00) |
| 1 | -0.02 (-0.17 to 0.13)*^P^* ^=.781^ | -0.01 (-0.08 to 0.07)*^P^* ^=.905^ | 0.05 (-0.02 to 0.11)*^P^* ^=.138^ | -0.00 (-0.03 to 0.02)*^P^* ^=.845^ | 0.00 (-0.01 to 0.01)*^P^* ^=.998^ | Ref. | -0.02 (-0.04 to 0.00)*^P^* ^=.083^ | . |
| 2 | -0.02 (-0.17 to 0.12)*^P^* ^=.741^ | 0.03 (-0.04 to 0.10)*^P^* ^=.387^ | 0.07 (0.01 to 0.13)*^P^* ^=.021^ | 0.02 (-0.01 to 0.04)*^P^* ^=.194^ | 0.01 (-0.00 to 0.02)*^P^* ^=.182^ | Ref. | -0.00 (-0.03 to 0.02)*^P^* ^= .690^ | . |
| 3 | 0.03 (-0.11 to 0.16)^P =.708^ | 0.06 (-0.01 to 0.12)*^P^* ^=.109^ | 0.10 (0.05 to 0.16) | 0.04 (0.02 to 0.06) | 0.02 (0.01 to 0.03)*^P^* ^=.001^ | Ref. | 0.00 (-0.02 to 0.02)*^P^* ^=.915^ | . |
| 4 | 0.03 (-0.10 to 0.16)*^P^* ^=.678^ | 0.04 (-0.03 to 0.10)*^P^* ^=.262^ | 0.09 (0.04 to 0.15)*^P^* ^=.001^ | 0.03 (0.01 to 0.06)*^P^* ^=.001^ | 0.02 (0.01 to 0.03)*^P^* ^=.001^ | Ref. | -0.02 (-0.04 to -0.00)*^P^* ^=.028^ | . |
| 5 | 0.09 (-0.05 to 0.22)*^P^* ^=.201^ | 0.07 (0.00 to 0.13)*^P^* ^=.047^ | 0.10 (0.05 to 0.15) | 0.04 (0.02 to 0.06) | 0.02 (0.01 to 0.03) | Ref. | -0.02 (-0.04 to 0.00)*^P^* ^=.033^ | . |
| b GA - gestational age (completed weeks) | | | |  |  |  |  |  |
| The *P*-value is less than .001 if not otherwise denoted. | | | | |  |  |  |  |
| Model 1: Unadjusted Model | | |  |  |  |  |  |  |
| Model 2: Adjusted for the sex, birth year, and for maternal and paternal ages at the birth of the individual | | | | | | | | |
| Model 3: Adjusted as Model 2 + maternal and paternal highest attained education | | | | | | | |  |
| Model 4: Adjusted as Model 3 + BWSDS, gestational disorder(s), maternal smoking at pregnancy, maternal marital status at the birth of the individual, and birth order | | | | | | | | |
| Model 5: Adjusted as Model 4 + severe medical condition of the individual | | | | | | |  |  |

### Supplementary Table S7.

| **Supplementary Table S7.** The number of individuals ^a^ with a native language grade in mainstream education, and the variation in grades on native language at 16 yr of age according to GA category in mainstream education expressed as grade differences and as z-scores with 95% confidence intervals | | | | | | | | |
| --- | --- | --- | --- | --- | --- | --- | --- | --- |
| GA^b^ | 23-27 weeks | 28-31 weeks | 32-33 weeks | 34-36 weeks | 37-38 weeks | 39-41 weeks | 42 weeks | 23-42 weeks |
| n (%) ^a^ | 175 (98.3) | 699 (99.7) | 1,020 (99.6) | 7,535 (99.7) | 36,763 (99.6) | 153,363 (99.6) | 8,325 (99.5) | 207,880 (99.6) |
| **Grade differences** | | | | | | | | |
| Grade, mean (SD) | 7.79 (1.17) | 7.73 (1.22) | 7.79 (1.15) | 7.76 (1.17) | 7.77 (1.18) | 7.79 (1.17) | 7.76 (1.16) | 7.79 (1.17) |
| 1 | 0.00 (-0.17 to 0.18)*^P^* ^=.973^ | -0.07 (-0.15 to 0.02)^P =.145^ | -0.00 (-0.08 to 0.07)*^P^* ^=.933^ | -0.03 (-0.06 to -0.00)*^P^* ^=.027^ | -0.02 (-0.04 to -0.01)*^P^* ^=.002^ | Ref. | -0.03 (-0.05 to -0.00)*^P^* ^=.042^ | . |
| 2 | 0.02 (-0.14 to 0.17)*^P^* ^=.839^ | -0.00 (-0.08 to 0.08)*^P^* ^=.951^ | 0.04 (-0.03 to 0.10)*^P^* ^=.282^ | -0.00 (-0.03 to 0.02)*^P^* ^=.960^ | -0.00 (-0.02 to 0.01)*^P^* ^=.473^ | Ref. | -0.01 (-0.03 to 0.02)*^P^* ^=.574^ | . |
| 3 | 0.06 (-0.09 to 0.21)*^P^* ^=.412^ | 0.02 (-0.06 to -0.09)*^P^* ^=.615^ | 0.07 (0.01 to 0.13)*^P^* ^=.035^ | 0.03 (0.00 to 0.05)*^P^* ^=.028^ | 0.00 (-0.01 to 0.02)*^P^* ^=.481^ | Ref. | -0.00 (-0.02 to 0.02)*^P^* ^=.886^ | . |
| 4 | 0.07 (-0.08 to 0.21)*^P^* ^=.360^ | 0.00 (-0.07 to 0.08)*^P^* ^=.958^ | 0.06 (-0.00 to 0.12)*^P^* ^=.084^ | 0.02 (-0.01 to 0.04)*^P^* ^=.168^ | 0.00 (-0.01 to 0.02)*^P^* ^=.461^ | Ref. | -0.02 (-0.05 to -0.00)*^P^* ^=.039^ | . |
| 5 | 0.13 (-0.02 to 0.28)*^P^* ^=.088^ | 0.03 (-0.04 to 0.11)*^P^* ^=.396^ | 0.06 (0.00 to 0.12)*^P^* ^=.044^ | 0.02 (-0.00 to 0.04)*^P^* ^=.101^ | 0.01 (-0.01 to 0.02)*^P^* ^=.375^ | Ref. | -0.02 (-0.04 to 0.00)*^P^* ^=.047^ | . |
| **Z-scores** | | | | | | | | |
| Z-score, mean (SD) | 0.01 (1.00) | -0.05 (1.04) | 0.00 (0.99) | -0.02 (1.00) | -0.01 (1.01) | 0.01 (1.00) | -0.02 (0.99) | 0.00 (1.00) |
| 1 | 0.00 (-0.15 to 0.15)*^P^* ^=.973^ | -0.06 (-0.13 to 0.19)*^P^* ^=.145^ | -0.00 (-0.06 to 0.06)*^P^* ^=.933^ | -0.03 (-0.05 to -0.00)^P =.027^ | -0.02 (-0.03 to -0.01)*^P^* ^=.002^ | Ref. | -0.02 (-0.05 to -0.00)*^P^* ^=.042^ | . |
| 2 | 0.01 (-0.12 to 0.15)*^P^* ^=.839^ | -0.00 (-0.07 to 0.07)*^P^* ^=.951^ | 0.03 (-0.03 to 0.09)*^P^* ^=.282^ | -0.00 (-0.02 to 0.02)^P =.960^ | -0.00 (-0.01 to 0.01)*^P^* ^=.473^ | Ref. | -0.01 (-0.03 to 0.01)*^P^* ^=.574^ | . |
| 3 | 0.05 (-0.07 to 0.18)*^P^* ^=.412^ | 0.02 (-0.05 to 0.08)*^P^* ^=.615^ | 0.06 (0.00 to 0.11)*^P^* ^=.035^ | 0.02 (0.00 to 0.04)*^P^* ^=.028^ | 0.00 (-0.01 to 0.01)*^P^* ^=.481^ | Ref. | 0.01 (-0.02 to 0.02)*^P^* ^=.886^ | . |
| 4 | 0.06 (-0.07 to 0.19)*^P^* ^=.360^ | 0.00 (-0.06 to 0.07)*^P^* ^=.958^ | 0.05 (-0.01 to 0.01)*^P^* ^=.084^ | 0.01 (-0.01 to 0.03)*^P^* ^=.168^ | 0.00 (-0.01 to 0.01)*^P^* ^=.461^ | Ref. | -0.02 (-0.04 to -0.00)*^P^* ^=.039^ | . |
| 5 | 0.11 (-0.02 to 0.24)*^P^* ^=.088^ | 0.03 (-0.04 to 0.09)*^P^* ^=.396^ | 0.05 (0.00 to 0.11)*^P^* ^=.049^ | 0.02 (-0.00 to 0.04)*^P^* ^=.101^ | 0.00 (-0.01 to 0.01)*^P^* ^=.375^ | Ref. | -0.02 (-0.4 to 0.00)*^P^* ^=.047^ | . |
| b GA - gestational age (completed weeks) | | | |  |  |  |  |  |
| The *P*-value is less than .001 if not otherwise denoted. | | | | |  |  |  |  |
| Model 1: Unadjusted Model | | |  |  |  |  |  |  |
| Model 2: Adjusted for the sex, birth year, and for maternal and paternal ages at the birth of the individual | | | | | | | | |
| Model 3: Adjusted as Model 2 + maternal and paternal highest attained education | | | | | | |  |  |
| Model 4: Adjusted as Model 3 + BWSDS, gestational disorder(s), maternal smoking at pregnancy, maternal marital status at the birth of the individual, and birth order | | | | | | | | |
| Model 5: Adjusted as Model 4 + severe medical condition of the individual | | | | | | |  |  |

### Supplementary Table S8.

| **Supplementary Table S8.** The number of individuals ^a^ with a physical education grade in mainstream education, and the variation in grades in physical education at 16 yr of age according to GA category in mainstream education expressed as grade differences and as z-scores with 95% confidence intervals | | | | | | | | |
| --- | --- | --- | --- | --- | --- | --- | --- | --- |
| GA^b^ | 23-27 weeks | 28-31 weeks | 32-33 weeks | 34-36 weeks | 37-38 weeks | 39-41 weeks | 42 weeks | 23-42 weeks |
| n (%) ^a^ | 170 (95.5) | 690 (98.4) | 1,012 (98.8) | 7,512 (99.4) | 36,713 (99.5) | 153 161 (99.5) | 8,311 (99.3) | 207,569 (99.5) |
| **Grade differences** | | | | | | | | |
| Grade, mean (SD) | 7.85 (0.93) | 8.11 (1.02) | 8.17 (1.03) | 8.24 (1.03) | 8.27 (1.04) | 8.27 (1.04) | 8.23 (1.06) | 8.27 (1.04) |
| 1 | -0.42 (-0.58 to -0.27) | -0.17 (-0.24 to -0.09) | -0.11 (-0.17 to -0.04)*^P^* ^=.001^ | -0.04 (-0.06 to -0.01)*^P^* ^=.002^ | -0.01 (-0.02 to 0.00)*^P^* ^=.124^ | Ref. | -0.05 (-0.07 to -0.02) | . |
| 2 | -0.41 (-0.57 to -0.25) | -0.16 (-0.22 to -0.07) | -0.09 (-0.16 to -0.03)*^P^* ^=.005^ | -0.03 (-0.05 to -0.00)*^P^* ^=.026^ | -0.00 (-0.02 to 0.01)*^P^* ^=.526^ | Ref. | -0.04 (-0.07 to -0.02) | . |
| 3 | -0.37 (-0.53 to -0.22) | -0.13 (-0.21 to -0.06)*^P^* ^=.001^ | -0.07 (-0.14 to -0.01)*^P^* ^=.024^ | -0.01 (-0.04 to 0.01)*^P^* ^=.328^ | 0.00 (-0.01 to 0.01)*^P^* ^=.750^ | Ref. | -0.04 (-0.06 to -0.02)*^P^* ^=.001^ | . |
| 4 | -0.34 (-0.49 to -0.19) | -0.12 (-0.19 to -0.04)*^P^* ^=.002^ | -0.05 (-0.11 to 0.01)*^P^* ^=.115^ | -0.00 (-0.02 to 0.02)*^P^* ^=.995^ | 0.01 (-0.00 to 0.02)*^P^* ^=.202^ | Ref. | -0.04 (-0.06 to -0.01)*^P^* ^=.002^ | . |
| 5 | -0.27 (-0.42 to -0.12)*^P^* ^=.001^ | -0.08 (-0.16 to -0.01)*^P^* ^=.032^ | -0.04 (-0.10 to 0.02)*^P^* ^=.212^ | 0.00 (-0.02 to 0.03)*^P^* ^=.767^ | 0.01 (-0.00 to 0.02)*^P^* ^=.140^ | Ref. | -0.03 (-0.06 to -0.01)*^P^* ^=.003^ | . |
| **Z-scores** | | | | | | | | |
| Z-score, mean (SD) | -0.40 (0.89) | -0.15 (0.98) | -0.10 (0.99) | -0.03 (0.99) | 0.00 (1.00) | 0.01 (1.00) | -0.04 (1.02) | 0.00 (1.00) |
| 1 | -0.41 (-0.56 to -0.26) | -0.16 (-0.23 to -0.08) | -0.10 (-0.17 to -0.04)*^P^* ^=.001^ | -0.04 (-0.06 to -0.01)*^P^* ^=.002^ | -0.01 (-0.02 to 0.00)*^P^* ^=.124^ | Ref. | -0.04 (-0.07 to -0.02) | . |
| 2 | -0.39 (-0.54 to -0.24) | -0.14 (-0.21 to -0.07) | -0.09 (-0.15 to -0.03)*^P^* ^=.005^ | -0.03 (-0.05 to -0.00)*^P^* ^=.026^ | -0.00 (-0.02 to 0.01)*^P^* ^=.526^ | Ref. | -0.04 (-0.06 to -0.02) | . |
| 3 | -0.36 (-0.51 to -0.21) | -0.13 (-0.20 to -0.06)*^P^* ^=.001^ | -0.07 (-0.13 to -0.01)*^P^* ^=.024^ | -0.01 (-0.03 to -0.01)*^P^* ^=.328^ | 0.00 (-0.01 to 0.01)*^P^* ^=.750^ | Ref. | -0.04 (-0.06 to -0.02)*^P^* ^=.001^ | . |
| 4 | -0.33 (-0.47 to -0.18) | -0.11 (-0.19 to -0.04)*^P^* ^=.002^ | -0.05 (-0.11 to 0.01)*^P^* ^=.115^ | -0.00 (-0.02 to 0.02)*^P^* ^=.995^ | 0.01 (-0.00 to 0.02)*^P^* ^=.202^ | Ref. | -0.03 (-0.06 to -0.01)*^P^* ^=.002^ | . |
| 5 | -0.26 (-0.41 to -0.11)*^P^*^=.001^ | -0.08 (-0.15 to -0.01)*^P^* ^=.032^ | -0.04 (-0.10 to 0.01)*^P^* ^=.212^ | 0.00 (-0.02 to 0.03)*^P^* ^=.767^ | 0.01 (-0.00 to -0.02)*^P^* ^=.140^ | Ref. | -0.03 (-0.05 to -0.01)*^P^* ^=.003^ | . |
| b GA - gestational age (completed weeks) | | | |  |  |  |  |  |
| The *P*-value is less than .001 if not otherwise denoted. | | | | |  |  |  |  |
| Model 1: Unadjusted Model | | |  |  |  |  |  |  |
| Model 2: Adjusted for the sex, birth year, and for maternal and paternal ages at the birth of the individual | | | | | | | | |
| Model 3: Adjusted as Model 2 + maternal and paternal highest attained education | | | | | | | |  |

| Model 4: Adjusted as Model 3 + BWSDS, gestational disorder(s), maternal smoking at pregnancy, maternal marital status at the birth of the individual, and birth order  Model 5: Adjusted as Model 4 + severe medical condition of the individual |
| --- |

### Supplementary Table S9ab.

| **Supplementary Table S9a.** Number of individuals with **low education (basic only or unknown education) at 25 years of age** according to GA category, and Odds Ratios (OR) with 95% Confidence Intervals for basic only or unknown education. Only such individuals who attended mainstream education in compulsory education are included. | | | | | | | | |
| --- | --- | --- | --- | --- | --- | --- | --- | --- |
| GA^a^ | 23-27 weeks | 28-31 weeks | 32-33 weeks | 34-36 weeks | 37-38 weeks | 39-41 weeks | 42 weeks | Total |
| n | 177 | 698 | 1,017 | 7,526 | 36,766 | 153,456 | 8,337 | 207,977 |
| basic only or unknown education, n (%) | 20 (11.3) | 76 (10.9) | 96 (9.4) | 857 (11.4) | 3,965 (10.8) | 16,276 (10.6) | 1,014 (12.2) | 22,204 (10.7) |
| Model 1 | 0.96 (0.60-1.54)*^P^* ^=.863^ | 0.99 (0.77-1.26)*^P^* ^=.901^ | 0.88 (0.71-1.09)*^P^* ^=.876^ | 1.07 (0.99-1.15)*^P^* ^=.098^ | 1.02 (0.98-1.06)*^P^* ^=.327^ | Ref. | 1.13 (1.06-1.21) | . |
| Model 2 | 0.99 (0.62-1.59)*^P^* ^=.972^ | 0.95 (0.75-1.22)*^P^* ^=.701^ | 0.85 (0.68-1.05)*^P^* ^=.136^ | 1.05 (0.97-1.13)*^P^* ^=.230^ | 1.02 (0.98-1.06)*^P^* ^=.361^ | Ref. | 1.11 (1.04-1.19)*^P^* ^=.003^ | . |
| Model 3 | 0.91 (0.56-1.47)*^P^* ^=.703^ | 0.94 (0.73-1.20)*^P^* ^=.620^ | 0.80 (0.64-0.99)*^P^* ^=.044^ | 1.01 (0.94-1.09)*^P^* ^=.785^ | 1.00 (0.96-1.04)*^P^* ^=.922^ | Ref. | 1.11 (1.03-1.19)*^P^* ^=.004^ | . |
| Model 4 | 0.86 (0.53-1.39)*^P^* ^=.541^ | 0.92 (0.71-1.17)*^P^* ^=.484^ | 0.77 (0.62-0.96)*^P^* ^=.018^ | 0.99 (0.92-1.07)*^P^* ^=.780^ | 1.00 (0.95-1.03)*^P^* ^=.988^ | Ref. | 1.12 (1.04-1.20)*^P^* ^=.003^ | . |
| Model 5 | 0.76 (0.47-1.24)*^P^* ^=.275^ | 0.86 (0.67-1.10)*^P^* ^=.225^ | 0.75 (0.60-0.94)*^P^* ^=.011^ | 0.98 (0.91-1.06)*^P^* ^=.663^ | 0.99 (0.95-1.02)*^P^* ^=.453^ | Ref. | 1.11 (1.04-1.20)*^P^* ^=.003^ | . |
|  |  |  |  |  |  |  |  |  |
|  |  |  |  |  |  |  |  |  |
| **Supplementary Table S9b.** Number of individuals with **high education (lower tertiary or higher education) at 25 years of age** according to GA category, and Odds Ratios (OR) with 95% Confidence Intervals for lower tertiary or higher education. Only such individuals who attended mainstream education in compulsory education are included. | | | | | | | | |
| GA^a^ | 23-27 weeks | 28-31 weeks | 32-33 weeks | 34-36 weeks | 37-38 weeks | 39-41 weeks | 42 weeks | Total |
| n | 177 | 698 | 1,017 | 7,526 | 36,766 | 153,456 | 8,337 | 207,977 |
| basic only or unknown education, n (%) | 33 (18.6) | 163 (23.4) | 269 (26.5) | 1,883 (25.0) | 9,660 (26.3) | 40,386 (26.3) | 1,994 (23.9) | 54,388 (26.2) |
| Model 1 | 0.64 (0.43-0.94)*^P^* ^=.022^ | 0.85 (0.71-1.02)*^P^* ^=.078^ | 0.99 (0.86-1.14)*^P^* ^=.877^ | 0.94 (0.89-1.00)*^P^* ^=.035^ | 1.00 (0.97-1.03)*^P^* ^=.971^ | Ref. | 0.90 (0.85-0.95) | . |
| Model 2 | 0.64 (0.43-0.94)*^P^* ^=.024^ | 0.90 (0.75-1.08)*^P^* ^=.264^ | 1.03 (0.89-1.19)*^P^* ^=.683^ | 0.97 (0.92-1.03)*^P^* ^=.280^ | 1.02 (0.99-1.04)*^P^* ^=.239^ | Ref. | 0.91 (0.86-0.96)*^P^* ^=.001^ | . |
| Model 3 | 0.65 (0.44-0.97)*^P^* ^=.036^ | 0.99 (0.77-1.11)*^P^* ^=.877^ | 1.08 (0.93-1.26)*^P^* ^=.290^ | 1.00 (0.95-1.06)*^P^* ^=.952^ | 1.03 (1.00-1.06)*^P^* ^=.041^ | Ref. | 0.91 (0.86-0.96)*^P^* ^=.001^ | . |
| Model 4 | 0.66 (0.45-0.99)*^P^* ^=.044^ | 0.92 (0.76-1.10)*^P^* ^=.354^ | 1.09 (0.94-1.27)*^P^* ^=.245^ | 1.00 (0.94-1.06)*^P^* ^=.991^ | 1.03 (1.00-1.06)*^P^* ^=.026^ | Ref. | 0.90 (0.85-0.95) | . |
| Model 5 | 0.73 (0.49-1.09)*^P^* ^=.126^ | 0.96 (0.80-1.16)*^P^* ^=.678^ | 1.11 (0.96-1.29)*^P^* ^=.170^ | 1.01 (0.95-1.06)*^P^* ^=.867^ | 1.03 (1.01-1.06)*^P^* ^=.023^ | Ref. | 0.90 (0.85-0.95) | . |
|  |  |  |  |  |  |  |  |  |
|  |  |  |  |  |  |  |  |  |
| a GA - gestational age (completed weeks) | | | | |  |  |  |  |
| The *P*-value is less than .001 if not otherwise denoted. | | | | | |  |  |  |
| Model 1: Unadjusted Model | | |  |  |  |  |  |  |
| Model 2: Adjusted for the sex, birth year, and for maternal and paternal ages at the birth of the individual | | | | | | | | |
| Model 3: Adjusted as Model 2 + maternal and paternal highest attained education | | | | | | | |  |
| Model 4: Adjusted as Model 3 + BWSDS, gestational disorder(s), maternal smoking at pregnancy, maternal marital status at the birth of the individual, and birth order | | | | | | | | |
| Model 5: Adjusted as Model 4 + severe medical condition of the individual | | | | | | | |  |

### Supplementary Table S10ab.

| **Supplementary Table S10a.** Odds Ratios and 95% Confidence Intervals for **low education (basic or unknown education) at 25 years of age** according to gestational age category and grade category in **mathematics** at 16 years of age as compared to secondary, less than tertiary education. Gestational age 39-41 weeks and grade category 7 to 8 serves as a reference (OR = 1.0). Only such individuals who attended mainstream education in compulsory education are included. | | | | | | |
| --- | --- | --- | --- | --- | --- | --- |
| Grade | Model | 23-33 weeks | 34-36 weeks | 37-38 weeks | 39-41 weeks | 42 weeks |
|  | 1 | 2.48 (2.01-3.06) | 2.95 (2.65-3.28) | 2.68 (2.53-2.83) | 2.62 (2.52-2.71) | 2.98 (2.69-3.30) |
| 4 | 2 | 2.37 (1.92-2.93) | 2.86 (2.57-3.18) | 2.64 (2.50-2.79) | 2.57 (2.48-2.66) | 2.91 (2.63-3.22) |
| to | 3 | 2.13 (1.72-2.64) | 2.59 (2.33-2.89) | 2.40 (2.27-2.54) | 2.38 (2.30-2.47) | 2.67 (2.43-2.98) |
| 6 | 4 | 2.01 (1.62-2.49) | 2.44 (2.19-2.73) | 2.32 (2.19-2.45) | 2.32 (2.24-2.41) | 2.60 (2.35-2.88) |
|  | 5 | 1.92 (1.55-2.39) | 2.42 (2.17-2.70) | 2.30 (2.18-2.44) | 2.32 (2.23-2.40) | 2.59 (2.34-2.87) |
|  | 1 | 0.86 (0.66-1.11)*^P^* ^=.232^ | 1.03 (0.91-1.16)*^P^* ^=.665^ | 1.00 (0.94-1.07)*^P^* ^=.966^ | Ref. | 1.16 (1.04-1.30)*^P^* ^=.008^ |
| 7 | 2 | 0.84 (0.65-1.09)*^P^* ^=.195^ | 1.01 (0.89-1.14)*^P^* ^=.878^ | 1.00 (0.94-1.06)*^P^* ^=.992^ |  | 1.14 (1.02-1.27)*^P^* ^=.021^ |
| to | 3 | 0.82 (0.64-1.06)*^P^* ^=.136^ | 0.98 (0.87-1.11)*^P^* ^=.753^ | 0.99 (0.93-1.05)*^P^* ^=.699^ |  | 1.13 (1.02-1.27)*^P^* ^=.024^ |
| 8 | 4 | 0.79 (0.61-1.02)*^P^* ^=.073^ | 0.97 (0.85-1.10)*^P^* ^=.597^ | 0.98 (0.92-1.04)*^P^* ^=.506^ |  | 1.14 (1.02-1.28)*^P^* ^=.020^ |
|  | 5 | 0.76 (0.59-0.98)*^P^* ^=.037^ | 0.96 (0.85-1.09)*^P^* ^=.534^ | 0.98 (0.92-1.04)*^P^* ^=.473^ |  | 1.14 (1.02-1.28)*^P^* ^=.023^ |
|  | 1 | 0.24 (0.11-0.51) | 0.41 (0.31-0.54) | 0.49 (0.43-0.55) | 0.46 (0.44-0.49) | 0.48 (0.38-0.61) |
| 9 | 2 | 0.24 (0.11-0.51) | 0.41 (0.31-0.54) | 0.49 (0.44-0.55) | 0.47 (0.44-0.50) | 0.48 (0.38-0.61) |
| to | 3 | 0.25 (0.12-0.53) | 0.43 (0.33-0.57) | 0.52 (0.46-0.59) | 0.51 (0.47-0.54) | 0.51 (0.40-0.65) |
| 10 | 4 | 0.25 (0.12-0.54) | 0.44 (0.33-0.58) | 0.53 (0.47-0.60) | 0.51 (0.48-0.55) | 0.52 (0.41-0.66) |
|  | 5 | 0.25 (0.12-0.52) | 0.44 (0.33-0.58) | 0.53 (0.47-0.60) | 0.52 (0.48-0.55) | 0.52 (0.41-0.66) |
|  |  |  |  |  |  |  |
| **Supplementary Table S10b.** Odds Ratios and 95% Confidence Intervals for **high education (lower tertiary or more) at 25 years of age** according to gestational age category and grade category in **mathematics** at 16 years of age as compared to secondary, less than tertiary education. Gestational age 39-41 weeks and grade category 7 to 8 serves as a reference (OR = 1.0). Only such individuals who attended mainstream education in compulsory education are included. | | | | | | |
| Grade | Model | 23-33 weeks | 34-36 weeks | 37-38 weeks | 39-41 weeks | 42 weeks |
|  | 1 | 0.14 (0.99-0.22) | 0.14 (0.11-0.18) | 0.18 (0.16-0.20) | 0.17 (0.16-0.18) | 0.16 (0.13-0.20) |
| 4 | 2 | 0.15 (0.10-0.24) | 0.15 (0.12-0.19) | 0.19 (0.18-0.21) | 0.18 (0.17-0.19) | 0.17 (0.14-0.21) |
| to | 3 | 0.17 (0.11-0.27) | 0.17 (0.13-0.21) | 0.21 (0.19-0.23) | 0.20 (0.19-0.21) | 0.19 (0.15-0.23) |
| 6 | 4 | 0.17 (0.11-0.28) | 0.17 (0.14-0.22) | 0.22 (0.20-0.24) | 0.20 (0.19-0.21) | 0.19 (0.15-0.24) |
|  | 5 | 0.18 (0.11-0.29) | 0.17 (0.14-0.22) | 0.20 (0.19-0.21) | 0.20 (0.19-0.21) | 0.19 (0.15-0.24) |
|  | 1 | 0.97 (0.83-1.14)*^P^* ^=.729^ | 0.95 (0.88-1.03)*^P^* ^=.218^ | 0.99 (0.95-1.03)*^P^* ^=.602^ | Ref. | 0.89 (0.82-0.96)*^P^* ^=.002^ |
| 7 | 2 | 1.02 (0.88-1.20)*^P^* ^=.767^ | 0.98 (0.91-1.07)*^P^* ^=.676^ | 1.00 (0.96-1.05)*^P^* ^=.868^ |  | 0.89 (0.82-0.97)*^P^* ^=.005^ |
| to | 3 | 1.05 (0.89-1.22)*^P^* ^=.584^ | 1.00 (0.92-1.09)*^P^* ^=.959^ | 1.01 (0.97-1.05)*^P^* ^=.547^ |  | 0.89 (0.82-0.97)*^P^* ^=.005^ |
| 8 | 4 | 1.06 (0.90-1.24)*^P^* ^=.493^ | 1.00 (0.92-1.09)*^P^* ^=.983^ | 1.01 (0.97-1.06)*^P^* ^=.489^ |  | 0.88 (0.81-0.96)*^P^* ^=.002^ |
|  | 5 | 1.09 (0.93-1.28)*^P^* ^=.297^ | 1.01 (0.93-1.09)*^P^* ^=.906^ | 1.02 (0.98-1.06)*^P^* ^=.472^ |  | 0.88 (0.81-0.96)*^P^* ^=.003^ |
|  | 1 | 2.55 (2.12-3.07) | 2.52 (2.31-2.75) | 2.70 (2.59-2.82) | 2.69 (2.62-2.76) | 2.40 (2.20-2.61) |
| 9 | 2 | 2.58 (2.13-3.11) | 2.58 (2.36-2.83) | 2.76 (2.64-2.89) | 2.71 (2.64-2.78) | 2.49 (2.28-2.72) |
| to | 3 | 2.42 (2.00-2.93) | 2.43 (2.22-2.66) | 2.56 (2.45-2.68) | 2.50 (2.43-2.57) | 2.31 (2.16-2.52) |
| 10 | 4 | 2.39 (1.97-2.90) | 2.38 (2.17-2.61) | 2.52 (2.41-2.64) | 2.46 (2.39-2.52) | 2.25 (2.06-2.46) |
|  | 5 | 2.42 (2.00-2.94) | 2.38 (2.17-2.61) | 2.52 (2.41-2.63) | 2.45 (2.39-2.52) | 2.25 (2.06-2.46) |

| a GA - gestational age (completed weeks) |
| --- |
| The *P*-value is less than .001 if not otherwise denoted. |
| Model 1: Unadjusted Model |
| Model 2: Adjusted for the sex, birth year, and for maternal and paternal ages at the birth of the individual |
| Model 3: Adjusted as Model 2 + maternal and paternal highest attained education |
| Model 4: Adjusted as Model 3 + BWSDS, gestational disorder(s), maternal smoking at pregnancy, maternal marital status at the birth of the individual, and birth order |
| Model 5: Adjusted as Model 4 + severe medical condition of the individual  The *p*-values (for Model 5) from the comparisons of interaction- and main-effect- models were .721 for grade 4-6 group; .638 for grade 7-8 group; and .718 for grade 9-10 group. For unadjusted model (Model 1) the *p*-values from the comparisons were as follows: .599 for grade 4-6 group; .785 for grade 7-8 group; and .704 for grade 9-10 group. |

### Supplementary Table S11ab.

| **Supplementary Table S11a.** Odds Ratios and 95% Confidence Intervals for **low education (basic or unknown education) at 25 years of age** according to gestational age category and grade category in **theoretical subjects** at 16 years of age as compared to secondary, less than tertiary education. Gestational age 39-41 weeks and grade category 7 to 8 serves as a reference (OR = 1.0). Only such individuals who attended mainstream education in compulsory education are included. | | | | | | | |
| --- | --- | --- | --- | --- | --- | --- | --- |
| Grade | Model | 23-33 weeks | 34-36 weeks | 37-38 weeks | 39-41 weeks | 42 weeks |  |
|  | 1 | 3.27 (2.65-4.03) | 3.86 (3.49-4.27) | 3.52 (3.34-3.71) | 3.40 (3.28-3.52) | 3.87 (3.51-4.26) | |
| 4 | 2 | 3.22 (2.61-3.97) | 3.79 (3.35-3.73) | 3.54 (3.35-3.73) | 3.40 (3.28-3.53) | 3.84 (3.48-4.24) | |
| to | 3 | 2.84 (2.30-3.52) | 3.39 (3.06-3.77) | 3.18 (3.01-3.36) | 3.12 (3.01-3.36) | 3.51 (3.18-3.88) | |
| 6 | 4 | 2.66 (2.15-3.30) | 3.18 (2.86-3.53) | 3.04 (2.88-3.22) | 3.03 (2.91-3.14) | 3.37 (3.05-3.72) | |
|  | 5 | 2.56 (2.07-3.17) | 3.16 (2.84-3.50) | 3.02 (2.86-3.20) | 3.01 (2.90-3.13) | 3.36 (3.04-3.71) |  |
|  | 1 | 0.88 (0.68-1.14)*^P^* ^=.336^ | 1.00 (0.88-1.13)*^P^* ^=.932^ | 0.99 (0.93-1.06)*^P^* ^=.773^ | Ref. | 1.15 (1.03-1.29)*^P^* ^=.013^ | |
| 7 | 2 | 0.87 (0.67-1.12)*^P^* ^=.271^ | 0.99 (0.87-1.12)*^P^* ^=.875^ | 0.99 (0.93-1.06)*^P^* ^=.853^ |  | 1.13 (1.01-1.27)*^P^* ^=.030^ | |
| to | 3 | 0.85 (0.65-1.10)*^P^* ^=.203^ | 0.97 (0.85-1.10)*^P^* ^=.609^ | 0.98 (0.92-1.05)*^P^* ^=.602^ |  | 1.13 (1.01-1.26)*^P^* ^=.034^ | |
| 8 | 4 | 0.81 (0.62-1.05)*^P^* ^=.108^ | 0.95 (0.84-1.08)*^P^* ^=.410^ | 0.98 (0.92-1.04)*^P^* ^=.474^ |  | 1.13 (1.01-1.26)*^P^* ^=.041^ | |
|  | 5 | 0.78 (0.60-1.01)*^P^* ^=.064^ | 0.94 (0.83-1.07)*^P^* ^=.371^ | 0.98 (0.92-1.04)*^P^* ^=.440^ |  | 1.12 (1.00-1.26)*^P^* ^=.045^ |  |
|  | 1 | 0.38 (0.14-1.03)*^P^* ^=.056^ | 0.42 (0.25-0.69)*^P^* ^=.001^ | 0.55 (0.45-0.67) | 0.53 (0.48-0.59) | 0.61 (0.41-0.90)*^P^* ^=.014^ | |
| 9 | 2 | 0.38 (0.14-1.03)*^P^* ^=.058^ | 0.42 (0.25-0.69)*^P^* ^=.001^ | 0.55 (0.45-0.67) | 0.53 (0.47-0.58) | 0.61 (0.41-0.90)*^P^* ^=.013^ | |
| to | 3 | 0.38 (0.14-1.04)*^P^* ^=.061^ | 0.44 (0.27-0.73)*^P^* ^=.002^ | 0.58 (0.48-0.71) | 0.57 (0.51-0.63) | 0.63 (0.43-0.94)*^P^* ^=.024^ | |
| 10 | 4 | 0.38 (0.14-1.04)*^P^* ^=.061^ | 0.45 (0.27-0.75)*^P^* ^=.002^ | 0.60 (0.49-0.73) | 0.58 (0.52-0.64) | 0.64 (0.43-0.95)*^P^* ^=.026^ | |
|  | 5 | 0.38 (0.14-1.02)^P =.055^ | 0.45 (0.27-0.75)*^P^* ^=.002^ | 0.60 (0.49-0.73) | 0.58 (0.52-0.64) | 0.64 (0.43-0.95)*^P^* ^=.038^ |  |
|  |  |  |  |  |  |  |  |
| **Supplementary Table S11b.** Odds Ratios and 95% Confidence Intervals for **high education (lower tertiary or more) at 25 years of age** according to gestational age category and grade category in **theoretical subjects** at 16 years of age as compared to secondary, less than tertiary education. Gestational age 39-41 weeks and grade category 7 to 8 serves as a reference (OR = 1.0). Only such individuals who attended mainstream education in compulsory education are included. | | | | | | | |
| Grade | Model | 23-33 weeks | 34-36 weeks | 37-38 weeks | 39-41 weeks | 42 weeks |  |
|  | 1 | 0.05 (0.03-0.10) | 0.05 (0.04-0.07) | 0.08 (0.07-0.09) | 0.07 (0.07-0.08) | 0.07 (0.05-0.09) | |
| 4 | 2 | 0.06 (0.03-0.12) | 0.06 (0.04-0.08) | 0.09 (0.08-0.10) | 0.08 (0.07-0.08) | 0.07 (0.06-0.10) | |
| to | 3 | 0.07 (0.04-0.13) | 0.07 (0.05-0.09) | 0.10 (0.09-0.11) | 0.09 (0.08-0.09) | 0.09 (0.07-0.11) | |
| 6 | 4 | 0.07 (0.04-0.14) | 0.07 (0.05-0.10) | 0.09 (0.08-0.10) | 0.09 (0.07-0.12) | 0.09 (0.07-0.12) | |
|  | 5 | 0.07 (0.04-0.14) | 0.07 (0.05-0.10) | 0.10 (0.09-0.12) | 0.09 (0.08-0.10) | 0.09 (0.07-0.12) |  |
|  | 1 | 0.89 (0.78-1.02)*^P^* ^=.498^ | 0.93 (0.87-1.00)*^P^* ^=.049^ | 0.99 (0.96-1.02)*^P^* ^=.498^ | Ref. | 0.90 (0.85-0.97)*^P^* ^=.002^ | |
| 7 | 2 | 0.92 (0.80-1.05)*^P^* ^=.227^ | 0.96 (0.90-1.03^)^*^P^* ^=.221^ | 1.00 (0.97-1.04)*^P^* ^=.947^ |  | 0.91 (0.85-0.97)*^P^* ^=.005^ | |
| to | 3 | 0.94 (0.82-1.08)*^P^* ^=.362^ | 0.98 (0.91-1.05)*^P^* ^=.540^ | 1.01 (0.98-1.04)*^P^* ^=.614^ |  | 0.91 (0.85-0.97)*^P^* ^=.005^ | |
| 8 | 4 | 0.96 (0.84-1.10)*^P^* ^=.573^ | 0.99 (0.92-1.06)*^P^* ^=.507^ | 1.01 (0.98-1.05)*^P^* ^=.507^ |  | 0.91 (0.85-0.97)*^P^* ^=.004^ | |
|  | 5 | 0.99 (0.86-1.13)*^P^* ^=.845^ | 0.99 (0.92-1.06)*^P^* ^=.770^ | 1.01 (0.98-1.05)*^P^* ^=.472^ |  | 0.91 (0.85-0.97)*^P^* ^=.004^ |  |
|  | 1 | 2.87 (2.24-3.68) | 3.15 (2.77-3.58) | 3.32 (3.13-3.52) | 3.33 (3.22-3.43) | 2.73 (2.41-3.10) | |
| 9 | 2 | 2.71 (2.11-3.49) | 2.97 (2.61-3.39) | 3.10 (2.92-3.29) | 3.06 (2.97-3.16) | 2.59 (2.28-2.93) | |
| to | 3 | 2.59 (2.01-3.33) | 2.75 (2.41-3.13) | 2.86 (2.69-3.03) | 2.81 (2.72-2.90) | 2.41 (2.12-2.73) | |
| 10 | 4 | 2.56 (1.99-3.30) | 2.69 (2.36-3.07) | 2.81 (2.64-2.98) | 2.76 (2.67-2.85) | 2.36 (2.08-2.68) | |
|  | 5 | 2.61 (2.03-3.37) | 2.69 (2.36-3.07) | 2.80 (2.63-2.97) | 2.75 (2.66-2.84) | 2.36 (2.08-2.68) |  |

| a GA - gestational age (completed weeks) |
| --- |
| The *P*-value is less than .001 if not otherwise denoted. |
| Model 1: Unadjusted Model |
| Model 2: Adjusted for the sex, birth year, and for maternal and paternal ages at the birth of the individual |
| Model 3: Adjusted as Model 2 + maternal and paternal highest attained education |
| Model 4: Adjusted as Model 3 + BWSDS, gestational disorder(s), maternal smoking at pregnancy, maternal marital status at the birth of the individual, and birth order |
| Model 5: Adjusted as Model 4 + severe medical condition of the individual  The *p*-values (for Model 5) from the comparisons of interaction- and main-effect- models were .316 for grade 4-6 group; .860 for grade 7-8 group; and .971 for grade 9-10 group. For unadjusted model (Model 1) the *p*-values from the comparisons were as follows: .220 for grade 4-6 group; .782 for grade 7-8 group; and .781 for grade 9-10 group. |

### Supplementary Table S12ab.

| **Supplementary Table S12a.** Odds Ratios and 95% Confidence Intervals for **low education (basic or unknown education) at 25 years of age** according to gestational age category and grade category in **native language** at 16 years of age as compared to secondary, less than tertiary education. Gestational age 39-41 weeks and grade category 7 to 8 serves as a reference (OR = 1.0). Only such individuals who attended mainstream education in compulsory education are included. | | | | | | |
| --- | --- | --- | --- | --- | --- | --- |
| Grade | Model | 23-33 weeks | 34-36 weeks | 37-38 weeks | 39-41 weeks | 42 weeks |
|  | 1 | 2.32 (1.76-3.06) | 3.26 (2.87-3.71) | 2.82 (2.65-3.00) | 2.89 (2.79-3.00) | 3.24 (2.86-3.67) |
| 4 | 2 | 2.54 (1.92-3.36) | 3.54 (3.11-4.03) | 3.16 (2.97-3.38) | 3.22 (3.09-3.34) | 3.59 (3.16-4.06) |
| to | 3 | 2.26 (1.70-3.00) | 3.17 (2.78-3.61) | 2.86 (2.68-3.05) | 2.96 (2.85-3.08) | 3.31 (2.92-3.75) |
| 6 | 4 | 2.11 (1.59-2.81) | 3.01 (2.63-3.43) | 2.73 (2.56-2.92) | 2.87 (2.76-2.99) | 3.19 (2.81-3.62) |
|  | 5 | 2.04 (1.53-2.71) | 2.97 (2.61-3.40) | 2.72 (2.54-2.90) | 2.86 (2.75-2.98) | 3.17 (2.79-3.60) |
|  | 1 | 1.00 (0.82-1.23)*^P^* ^=.998^ | 1.04 (0.94-1.15)*^P^* ^=.451^ | 1.03 (0.98-1.09)*^P^* ^=.245^ | Ref. | 1.17 (1.07-1.29)*^P^* ^=.001^ |
| 7 | 2 | 0.99 (0.81-1.22)*^P^* ^=.946^ | 1.04 (0.94-1.15)*^P^* ^=.474^ | 1.04 (0.99-1.09)*^P^* ^=.159^ |  | 1.15 (1.05-1.27)*^P^* ^=.003^ |
| to | 3 | 0.96 (0.78-1.19)*^P^* ^=.725^ | 1.01 (0.91-1.13)*^P^* ^=.793^ | 1.02 (0.97-1.08)*^P^* ^=.382^ |  | 1.15 (1.04-1.26)*^P^* ^=.005^ |
| 8 | 4 | 0.93 (0.75-1.14)*^P^* ^=.466^ | 0.99 (0.89-1.10)*^P^* ^=.826^ | 1.01 (0.96-1.07)*^P^* ^=.604^ |  | 1.15 (1.04-1.26)*^P^* ^=.004^ |
|  | 5 | 0.89 (0.73-1.10)*^P^* ^=.287^ | 0.98 (0.89-1.09)*^P^* ^=.754^ | 1.01 (0.96-1.07)*^P^* ^=.673^ |  | 1.15 (1.04-1.26)*^P^* ^=.005^ |
|  | 1 | 0.42 (0.25-0.69)*^P^* ^=.001^ | 0.42 (0.33-0.55) | 0.45 (0.40-0.50) | 0.45 (0.43-0.48) | 0.48 (0.38-0.60) |
| 9 | 2 | 0.38 (0.23-0.63) | 0.39 (0.30-0.50) | 0.41 (0.37-0.46) | 0.41 (0.39-0.44) | 0.43 (0.34-0.54) |
| to | 3 | 0.40 (0.24-0.66) | 0.41 (0.32-0.53) | 0.44 (0.39-0.50) | 0.45 (0.42-0.48) | 0.46 (0.37-0.58) |
| 10 | 4 | 0.39 (0.23-0.65) | 0.42 (0.32-0.54) | 0.45 (0.40-0.51) | 0.46 (0.43-0.49) | 0.47 (0.37-0.59) |
|  | 5 | 0.38 (0.23-0.63) | 0.42 (0.32-0.54) | 0.45 (0.40-0.51) | 0.46 (0.43-0.49) | 0.47 (0.37-0.59) |
|  |  |  |  |  |  |  |
| **Supplementary Table S12b.** Odds Ratios and 95% Confidence Intervals for **high education (lower tertiary or more) at 25 years of age** according to gestational age category and grade category in **native language** at 16 years of age as compared to secondary, less than tertiary education. Gestational age 39-41 weeks and grade category 7 to 8 serves as a reference (OR = 1.0). Only such individuals who attended mainstream education in compulsory education are included. | | | | | | |
| Grade | Model | 23-33 weeks | 34-36 weeks | 37-38 weeks | 39-41 weeks | 42 weeks |
|  | 1 | 0.09 (0.04-0.22) | 0.09 (0.05-0.14) | 0.11 (0.09-0.13) | 0.09 (0.08-0.10) | 0.09 (0.06-0.14) |
| 4 | 2 | 0.10 (0.04-0.24) | 0.10 (0.06-0.15) | 0.12 (0.10-0.14) | 0.10 (0.09-0.11) | 0.10 (0.06-0.15) |
| to | 3 | 0.11 (0.05-0.27) | 0.11 (0.07-0.17) | 0.13 (0.11-0.16) | 0.11 (0.10-0.12) | 0.11 (0.07-0.17) |
| 6 | 4 | 0.12 (0.05-0.28) | 0.11 (0.07-0.18) | 0.14 (0.11-0.16) | 0.11 (0.10-0.13) | 0.11 (0.07-0.17) |
|  | 5 | 0.12 (0.05-0.29) | 0.11 (0.07-0.18) | 0.14 (0.11-0.17) | 0.11 (0.10-0.13) | 0.11 (0.07-0.17) |
|  | 1 | 0.95 (0.81-1.11)*^P^* ^=.527^ | 0.94 (0.87-1.02)*^P^* ^=.128^ | 1.01 (0.97-1.05)*^P^* ^=.596^ | Ref. | 0.91 (0.85-0.99)*^P^* ^=.031^ |
| 7 | 2 | 0.97 (0.83-1.14)*^P^* ^=.726^ | 0.96 (0.88-1.04)*^P^* ^=.267^ | 1.02 (0.98-1.06)*^P^* ^=.411^ |  | 0.92 (0.86-1.00)*^P^* ^=.049^ |
| to | 3 | 1.00 (0.85-1.17)*^P^* ^=.994^ | 0.98 (0.90-1.06)*^P^* ^=.573^ | 1.03 (0.99-1.07)*^P^* ^=.211^ |  | 0.93 (0.86-1.00)*^P^* ^=.063^ |
| 8 | 4 | 1.02 (0.87-1.19)*^P^* ^=.834^ | 0.98 (0.91-1.07)*^P^* ^=.690^ | 1.03 (0.99-1.07)*^P^* ^=.160^ |  | 0.92 (0.85-1.00)*^P^* ^=.041^ |
|  | 5 | 1.04 (0.89-1.22)*^P^* ^=.615^ | 0.99 (0.91-1.07)*^P^* ^=.774^ | 1.03 (0.99-1.07)*^P^* ^=.144^ |  | 0.92 (0.85-1.00)*^P^* ^=.043^ |
|  | 1 | 3.50 (2.95-4.14) | 3.85 (3.53-4.20) | 4.07 (3.90-4.24) | 4.02 (3.92-4.12) | 3.45 (3.18-3.75) |
| 9 | 2 | 3.23 (2.72-3.83) | 3.53 (3.23-3.85) | 3.72 (3.56-3.88) | 3.65 (3.55-3.75) | 3.19 (2.92-3.46) |
| to | 3 | 3.02 (2.54-3.59) | 3.26 (2.99-3.56) | 3.39 (3.25-3.54) | 3.31 (3.22-3.40) | 2.88 (2.62-3.13) |
| 10 | 4 | 3.00 (2.52-3.57) | 3.20 (2.93-3.50) | 3.32 (3.18-3.46) | 3.23 (3.15-3.32) | 2.82 (2.59-3.07) |
|  | 5 | 3.08 (2.59-3.67) | 3.20 (2.93-3.50) | 3.31 (3.17-3.45) | 3.22 (3.14-3.31) | 2.82 (2.59-3.06) |

| a GA - gestational age (completed weeks) |
| --- |
| The *P*-value is less than .001 if not otherwise denoted. |
| Model 1: Unadjusted Model |
| Model 2: Adjusted for the sex, birth year, and for maternal and paternal ages at the birth of the individual |
| Model 3: Adjusted as Model 2 + maternal and paternal highest attained education |
| Model 4: Adjusted as Model 3 + BWSDS, gestational disorder(s), maternal smoking at pregnancy, maternal marital status at the birth of the individual, and birth order |
| Model 5: Adjusted as Model 4 + severe medical condition of the individual  The *p*-values (for Model 5) from the comparisons of interaction- and main-effect- models were .537 for grade 4-6 group; .770 for grade 7-8 group; and .958 for grade 9-10 group. For unadjusted model (Model 1) the *p*-values from the comparisons were as follows: .535 for grade 4-6 group; .796 for grade 7-8 group; and .895 for grade 9-10 group. |

### Supplementary Table S13ab.

| **Supplementary Table S13a.** Odds Ratios and 95% Confidence Intervals for **low education (basic or unknown education) at 25 years of age** according to gestational age category and grade category in **physical education** at 16 years of age as compared to secondary, less than tertiary education. Gestational age 39-41 weeks and grade category 7 to 8 serves as a reference (OR = 1.0). Only such individuals who attended mainstream education in compulsory education are included. | | | | | | |
| --- | --- | --- | --- | --- | --- | --- |
| Grade | Model | 23-33 weeks | 34-36 weeks | 37-38 weeks | 39-41 weeks | 42 weeks |
|  | 1 | 2.69 (1.78-4.07) | 4.34 (3.48-5.41) | 3.80 (3.44-4.20) | 3.73 (3.54-3.93) | 3.97 (3.28-4.80) |
| 4 | 2 | 2.66 (1.75-4.04) | 4.25 (3.40-5.30) | 3.72 (3.36-4.12) | 3.69 (3.50-3.89) | 3.86 (3.18-4.68) |
| to | 3 | 2.41 (1.58-3.69) | 3.97 (3.17-4.96) | 3.44 (3.11-3.81) | 3.44 (3.26-3.63) | 3.65 (3.00-4.43) |
| 6 | 4 | 2.23 (1.46-3.42) | 3.72 (2.96-4.66) | 3.26 (2.94-3.62) | 3.30 (3.13-3.48) | 3.52 (2.89-4.28) |
|  | 5 | 2.14 (1.40-3.29) | 3.67 (2.93-4.60) | 3.24 (2.92-3.59) | 3.28 (3.11-3.46) | 3.49 (2.87-4.24) |
|  | 1 | 0.91 (0.76-1.11)*^P^* ^=.354^ | 1.07 (0.97-1.17)*^P^* ^=.190^ | 0.99 (0.95-1.04)*^P^* ^=.781^ | Ref. | 1.10 (1.00-1.20)*^P^* ^=.053^ |
| 7 | 2 | 0.89 (0.74-1.08)*^P^* ^=.245^ | 1.05 (0.95-1.15)*^P^* ^=.332^ | 0.99 (0.95-1.04)*^P^* ^=.798^ |  | 1.08 (0.98-1.18)*^P^* ^=.983^ |
| to | 3 | 0.85 (0.70-1.03)*^P^* ^=.103^ | 1.02 (0.92-1.12)*^P^* ^=.768^ | 0.97 (0.92-1.01)*^P^* ^=.271^ |  | 1.08 (0.98-1.18)*^P^* ^=.118^ |
| 8 | 4 | 0.83 (0.68-1.01)*^P^* ^=.057^ | 0.99 (0.90-1.09)*^P^* ^=.892^ | 0.97 (0.92-1.01)*^P^* ^=.159^ |  | 1.09 (0.99-1.20)*^P^* ^=.069^ |
|  | 5 | 0.80 (0.66-0.97)*^P^* ^=.023^ | 1.00 (0.90-1.09)*^P^* ^=.836^ | 0.96 (0.92-1.01)*^P^* ^=.138^ |  | 1.09 (0.99-1.20)*^P^* ^=.073^ |
|  | 1 | 0.41 (0.28-0.60) | 0.54 (0.46-0.63) | 0.58 (0.54-0.63) | 0.54 (0.52-0.56) | 0.65 (0.57-0.74) |
| 9 | 2 | 0.40 (0.27-0.59) | 0.53 (0.46-0.63) | 0.59 (0.55-0.63) | 0.54 (0.52-0.57) | 0.64 (0.56-0.73) |
| to | 3 | 0.42 (0.29-0.62) | 0.55 (0.47-0.64) | 0.62 (0.57-0.66) | 0.57 (0.55-0.60) | 0.67 (0.58-0.77) |
| 10 | 4 | 0.42 (0.29-0.62) | 0.56 (0.47-0.66) | 0.63 (0.58-0.67) | 0.59 (0.56-0.61) | 0.68 (0.60-0.78) |
|  | 5 | 0.42 (0.28-0.61) | 0.56 (0.47-0.66) | 0.63 (0.58-0.68) | 0.59 (0.57-0.61) | 0.69 (0.60-0.79) |
|  |  |  |  |  |  |  |
| **Supplementary Table S13b.** Odds Ratios and 95% Confidence Intervals for **high education (lower tertiary or more) at 25 years of age** according to gestational age category and grade category in **physical education** at 16 years of age as compared to secondary, less than tertiary education. Gestational age 39-41 weeks and grade category 7 to 8 serves as a reference (OR = 1.0). Only such study individuals who attended mainstream education in compulsory education are included. | | | | | | |
| Grade | Model | 23-33 weeks | 34-36 weeks | 37-38 weeks | 39-41 weeks | 42 weeks |
|  | 1 | 0.24 (0.10-0.59)*^P^* ^=.002^ | 0.27 (0.16-0.46) | 0.23 (0.18-0.30) | 0.19 (0.17-0.22) | 0.30 (0.19-0.45) |
| 4 | 2 | 0.25 (0.10-0.63)*^P^* ^=.003^ | 0.28 (0.17-0.48) | 0.24 (0.18-0.30) | 0.19 (0.17-0.22) | 0.31 (0.20-0.47) |
| to | 3 | 0.29 (0.12-0.72)*^P^* ^=.008^ | 0.32 (0.19-0.54) | 0.26 (0.20-0.33) | 0.21 (0.19-0.24) | 0.33 (0.21-0.51) |
| 6 | 4 | 0.29 (0.12-0.74)*^P^* ^=.009^ | 0.34 (0.20-0.57) | 0.27 (0.21-0.34) | 0.22 (0.19-0.25) | 0.34 (0.22-0.52) |
|  | 5 | 0.31 (0.12-0.77)^P =.012^ | 0.34 (0.20-0.58) | 0.27 (0.21-0.35) | 0.22 (0.20-0.25) | 0.34 (0.22-0.52) |
|  | 1 | 0.99 (0.86-1.15)*^P^* ^=.913^ | 0.94 (0.86-1.02)*^P^* ^=.108^ | 1.02 (0.98-1.06)*^P^* ^=.436^ | Ref. | 0.91 (0.84-0.98)*^P^* ^=.014^ |
| 7 | 2 | 1.04 (0.90-1.21)*^P^* ^=.583^ | 0.96 (0.89-1.05)*^P^* ^=.380^ | 1.03 (0.99-1.07)*^P^* ^=.140^ |  | 0.91 (0.84-0.99)*^P^* ^=.023^ |
| to | 3 | 1.07 (0.92-1.24)*^P^* ^=.411^ | 0.99 (0.91-1.08)*^P^* ^=.796^ | 1.05 (1.01-1.09)*^P^* ^=.023^ |  | 0.91 (0.84-0.98)*^P^* ^=.017^ |
| 8 | 4 | 1.06 (0.91-1.24)*^P^* ^=.453^ | 0.99 (0.91-1.07)*^P^* ^=.766^ | 1.05 (1.01-1.09)*^P^* ^=.020^ |  | 0.89 (0.82-0.96)*^P^* ^=.004^ |
|  | 5 | 1.10 (0.94-1.28)*^P^* ^=.237^ | 0.99 (0.91-1.08)*^P^* ^=.832^ | 1.05 (1.01-1.10)*^P^* ^=.017^ |  | 0.89 (0.82-0.96)*^P^* ^=.004^ |
|  | 1 | 1.94 (1.64-2.29) | 2.00 (1.85-2.16) | 2.05 (1.97-2.12) | 2.06 (2.01-2.10) | 1.83 (1.70-1.98) |
| 9 | 2 | 2.01 (1.70-2.38) | 2.05 (1.89-2.22) | 2.08 (2.01-2.17) | 2.06 (2.01-2.11) | 1.89 (1.75-2.04) |
| to | 3 | 1.93 (1.63-2.30) | 1.96 (1.81-2.12) | 1.94 (1.87-2.02) | 1.92 (1.87-1.96) | 1.77 (1.64-1.91) |
| 10 | 4 | 1.91 (1.61-2.27) | 1.91 (1.76-2.07) | 1.92 (1.84-1.99) | 1.89 (1.84-1.94) | 1.72 (1.59-1.86) |
|  | 5 | 1.92 (1.62-2.28) | 1.91 (1.76-2.07) | 1.91 (1.84-1.99) | 1.88 (1.84-1.93) | 1.71 (1.59-1.85) |

| a GA - gestational age (completed weeks) |
| --- |
| The *P*-value is less than .001 if not otherwise denoted. |
| Model 1: Unadjusted Model |
| Model 2: Adjusted for the sex, birth year of the individual, and for maternal and paternal ages at the birth of the individual |
| Model 3: Adjusted as Model 2 + maternal and paternal highest attained education |
| Model 4: Adjusted as Model 3 + BWSDS, gestational disorder(s), maternal smoking at pregnancy, maternal marital status at the birth of the individual, and birth order |
| Model 5: Adjusted as Model 4 + severe medical condition of the individual  The *p*-values (for Model 5) from the comparisons of interaction- and main-effect- models were .281 for grade 4-6 group; .473 for grade 7-8 group; and .319 for grade 9-10 group. For unadjusted model (Model 1) the *p*-values from the comparisons were as follows: .276 for grade 4-6 group; .589 for grade 7-8 group; and .424 for grade 9-10 group. |

## SUPPLEMENTARY FIGURES

## Supplementary Figure 1.


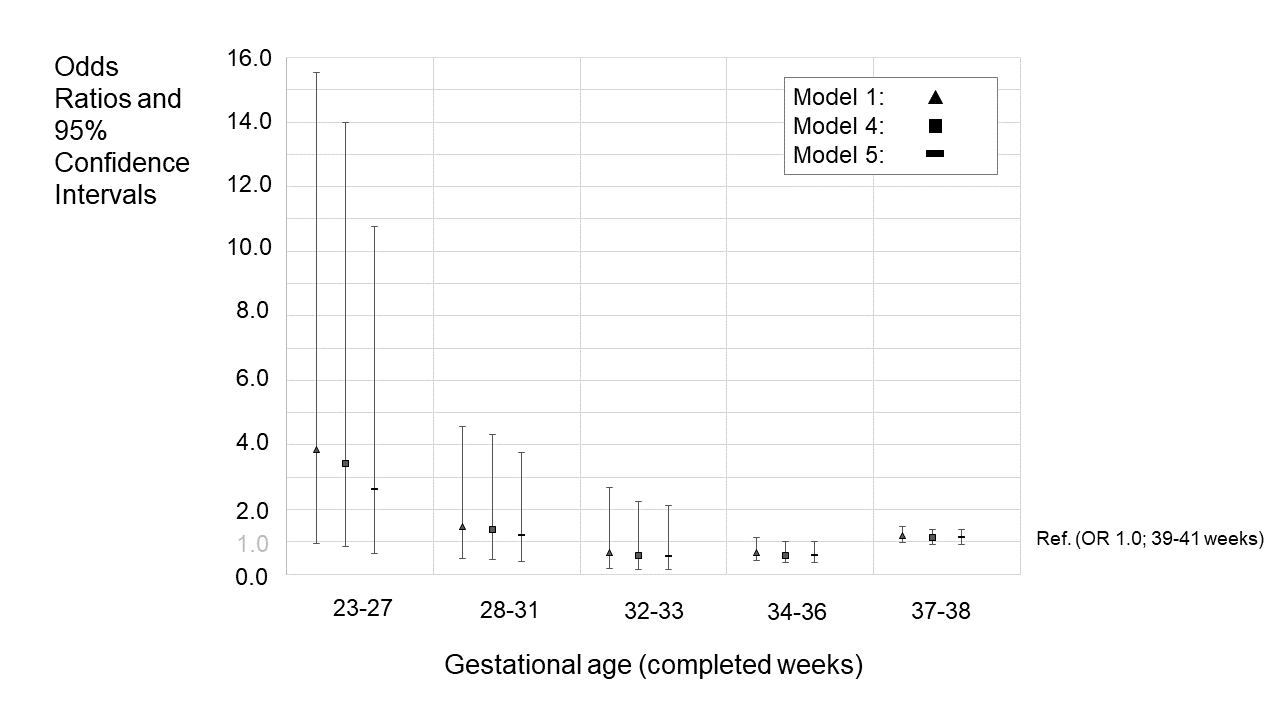


**Supplementary Figure 1.** Odds ratios (ORs) and 95% confidence intervals (CIs) for discontinuing education in compulsory education according to gestational age category. The figure shows models 1, 4, and 5. Models 2 and 3 are available in the Supplementary Table 4. GA category 39-41 weeks is the referent (OR= 1.0).

Model 1; Unadjusted model,

Model 4; Adjusted for the sex, birth year, maternal and paternal ages, maternal and paternal highest attained education, BWSDS, gestational disorder(s), maternal smoking at pregnancy, maternal marital status at the birth of the individual, and birth order,

Model 5; Adjusted as Model 4, and for severe medical condition

## Supplementary Figure 2.


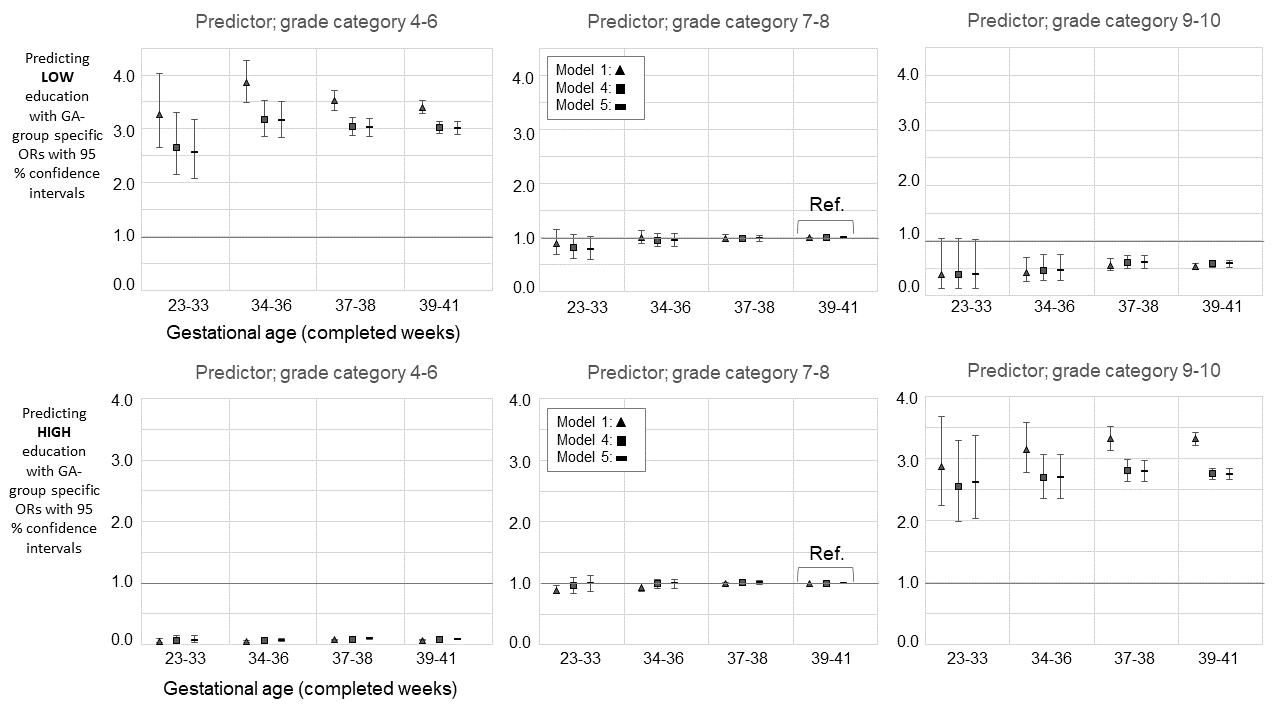


**Supplementary Figure 2.** The mean of theoretical subjects and gestational age category together predicting [LOW] ‘basic only or unknown’ (upper panel) and [HIGH] ‘lower tertiary or more’ (lower panel) education. Comparisons to intermediate education i.e., ‘upper secondary, less than tertiary’. GA 39-41 and grade category 7-8 serve as a reference group. The figures show models 1, 4, and 5. Models 2 and 3 are available in the Supplementary Table 11ab.The *p*-values (for Model 5) from the comparisons of interaction- and main-effect- models were .316 for grade 4-6 group; .860 for grade 7-8 group; and .971 for grade 9-10 group. For unadjusted model (Model 1) the *p*-values from the comparisons were as follows: .220 for grade 4-6 group; .782 for grade 7-8 group; and .781 for grade 9-10 group.

Model 1; Unadjusted model,

Model 4; Adjusted for the sex, birth year, maternal and paternal ages, maternal and paternal highest attained education, BWSDS, gestational disorder(s), maternal smoking at pregnancy, maternal marital status at the birth of the individual, and birth order,

Model 5; Adjusted as Model 4, and for severe medical condition

## Supplementary Figure 3.


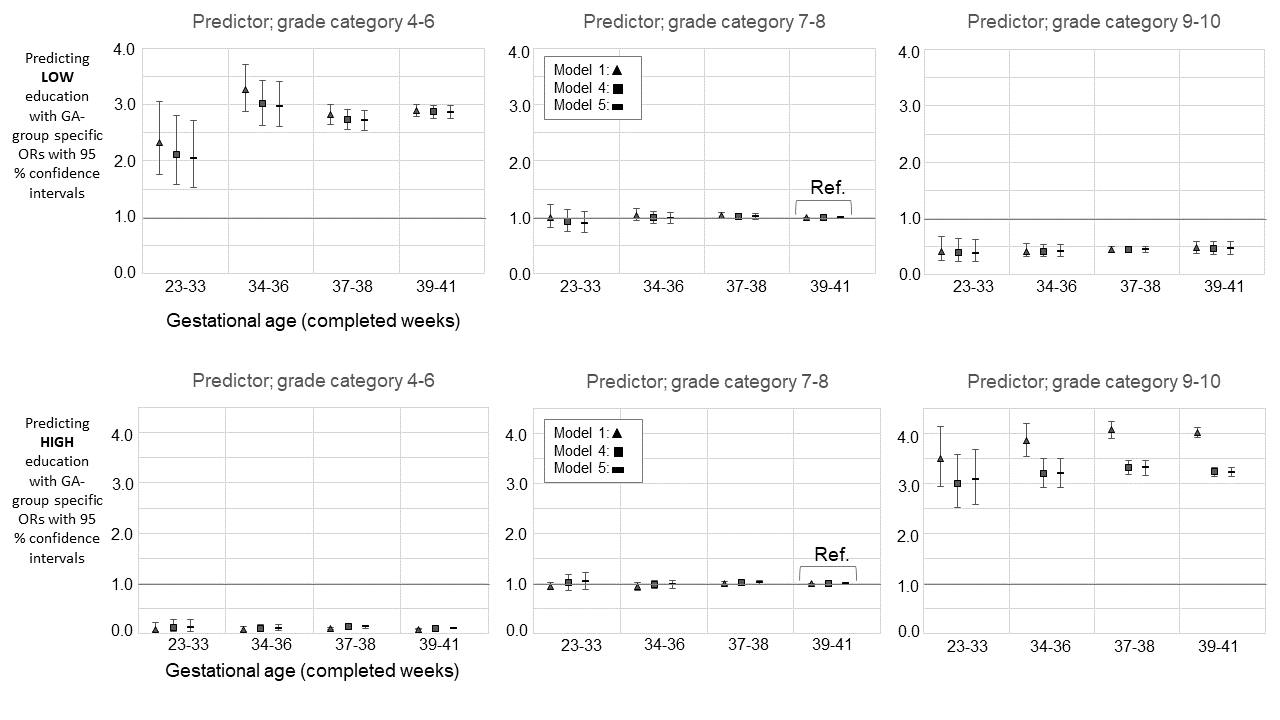


**Supplementary Figure 3.** Native language grade and gestational age category together predicting [LOW] ‘basic only or unknown’ (upper panel) and [HIGH] ‘lower tertiary or more’ (lower panel) education. Comparisons to intermediate education i.e., ‘upper secondary, less than tertiary’. GA 39-41 and grade category 7-8 serve as a reference group. The figures show models 1, 4, and 5. Models 2 and 3 are available in the Supplementary Table 12ab. The *p*-values (for Model 5) from the comparisons of interaction- and main-effect- models were .537 for grade 4-6 group; .770 for grade 7-8 group; and .958 for grade 9-10 group. For unadjusted model (Model 1) the *p*-values from the comparisons were as follows: .535 for grade 4-6 group; .796 for grade 7-8 group; and .895 for grade 9-10 group.

Model 1; Unadjusted model,

Model 4; Adjusted for the sex, birth year, maternal and paternal ages, maternal and paternal highest attained education, BWSDS, gestational disorder(s), maternal smoking at pregnancy, maternal marital status at the birth of the individual, and birth order,

Model 5; Adjusted as Model 4, and for severe medical condition

## Supplementary Figure 4.


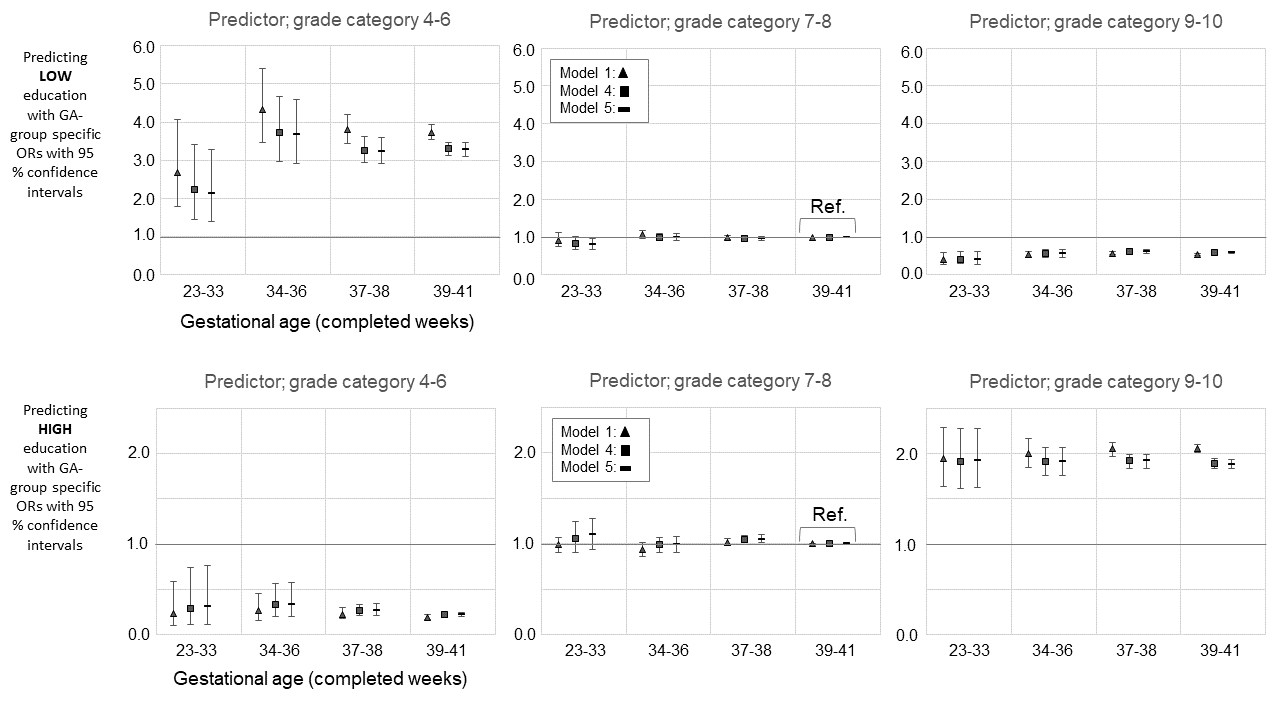


**Supplementary Figure 4.** Physical education grade and gestational age category together predicting [LOW] ‘basic only or unknown’ (upper panel) and [HIGH] ‘lower tertiary or more’ (lower panel) education. Comparisons to intermediate education i.e., ‘upper secondary, less than tertiary’. GA 39-41 and grade category 7-8 serve as a reference group. The figures show models 1, 4, and 5. Models 2 and 3 are available in the Supplementary Table 13ab. The *p*-values (for Model 5) from the comparisons of interaction- and main-effect- models were .281 for grade 4-6 group; .473 for grade 7-8 group; and .319 for grade 9-10 group. For unadjusted model (Model 1) the *p*-values from the comparisons were as follows: .276 for grade 4-6 group; .589 for grade 7-8 group; and .424 for grade 9-10 group.

Model 1; Unadjusted model,

Model 4; Adjusted for the sex, birth year, maternal and paternal ages, maternal and paternal highest attained education, BWSDS, gestational disorder(s), maternal smoking at pregnancy, maternal marital status at the birth of the individual, and birth order,

Model 5; Adjusted as Model 4, and severe medical condition
